# Contrasting sap flow characteristics between pioneer and late-successional tree species in secondary tropical montane forests of Eastern Himalaya, India

**DOI:** 10.1093/jxb/erad207

**Published:** 2023-06-08

**Authors:** Manish Kumar, Gladwin Joseph, Yangchenla Bhutia, Jagdish Krishnaswamy

**Affiliations:** Ashoka Trust for Research in Ecology and the Environment (ATREE), Bangalore 560064, Karnataka, India; Manipal Academy of Higher Education (MAHE), Manipal 576104, Karnataka, India; School of Geography, Earth and Environmental Sciences, University of Birmingham, Birmingham B15 2TT, UK; Ashoka Trust for Research in Ecology and the Environment (ATREE), Bangalore 560064, Karnataka, India; Conservation Biology Institute, Corvallis, Oregon 97333, USA; Ashoka Trust for Research in Ecology and the Environment (ATREE), Bangalore 560064, Karnataka, India; Manipal Academy of Higher Education (MAHE), Manipal 576104, Karnataka, India; Sikkim State Council of Science & Technology, Gangtok 737102, Sikkim, India; Ashoka Trust for Research in Ecology and the Environment (ATREE), Bangalore 560064, Karnataka, India; Manipal Academy of Higher Education (MAHE), Manipal 576104, Karnataka, India; School of Environment and Sustainability, Indian Institute for Human Settlements, Bangalore 560080, Karnataka, India; The Hebrew University of Jerusalem, Israel

**Keywords:** Ecohydrology, Himalaya, late-successional, pioneer species, plant water relations, sap flow, secondary forests, transpiration

## Abstract

The interactive role of life-history traits and environmental factors on plant water relations is crucial for understanding the responses of species to climate change, but it remains poorly understood in secondary tropical montane forests (TMFs). In this study, we examined differences in sap flow between the pioneer species *Symplocos racemosa* and *Eurya acuminata*, and the late-successional species *Castanopsis hystrix* that co-occur in a biodiverse Eastern Himalayan secondary broadleaved TMF. The fast-growing pioneers had sap flux densities that were 1.6–2.1 times higher than the late-successional species, and exhibited characteristics of long-lived pioneer species. Significant radial and azimuthal variability in sap flow (*V*) between species was observed and could be attributed to the life-history trait and the access of the canopy to sunlight. Nocturnal *V* was 13.8% of the daily total and was attributable to stem recharge during the evening period (18.00–23.00 h) and to endogenous stomatal controls during the pre-dawn period (00.00–05.00 h). The shallow-rooted pioneer species both exhibited midday depression in *V* that was attributable to photosensitivity and diel moisture stress responses. In contrast, the deep-rooted late-successional species showed unaffected transpiration across the dry season, indicating their access to groundwater. Thus, our results suggest that secondary broadleaved TMFs, with a dominance of shallow-rooted pioneers, are more prone to the negative impacts of drier and warmer winters than primary forests, which are dominated by deep-rooted species. Our study provides an empirical understanding of how life-history traits coupled with microclimate can modulate plant water use in the widely distributed secondary TMFs in Eastern Himalaya, and highlights their vulnerability to warmer winters and reduced winter precipitation due to climate change.

## Teaser

*In situ*sap-flow measurements from Eastern Himalayan secondary tropical montane forests suggest that co-occurring shallow-rooted pioneers are more vulnerable to drier and warmer winters than deep-rooted late-successional species.

## Graphical Abstract

**Figure:**
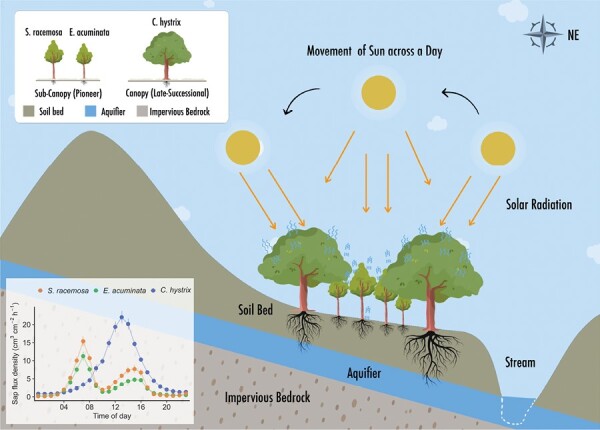
The access to soil moisture and sunlight dictates diurnal sap flux density (J in cm^3^ cm^-2^ h^-1^) patterns among co-occurring pioneer and emergent species in a secondary tropical montane forest in Eastern Himalaya.

## Introduction

Plant water relations play a crucial role in modulating carbon and water fluxes in recovering secondary tropical forests. In most terrestrial systems, the availability of energy and water are the primary drivers of water use by vegetation, mainly through transpiration (Heimann and Reichstein, 2008; Asbjornsen *et al.*, 2011). Transpiration is the movement of water from the soil to the atmosphere via the stem based on a gradient of total water potential along the soil–plant–atmosphere–continuum (SPAC), and is an active area of research in ecohydrology (Asbjornsen *et al.*, 2011; Manzoni *et al.*, 2013; Mencuccini *et al.*, 2019). Transpiration is driven by environmental variables such as vapour pressure deficit (VPD), light, and soil moisture availability, and by ecophysiological traits controlling plant water use such as wood anatomy, rooting depth, stomatal density, and sapwood density (Asbjornsen *et al.*, 2011; Manzoni *et al.*, 2013). These ecophysiological traits have evolved through competition and adaptation between co-occurring species belonging to different functional groups, such as pioneer or late-successional, and they contribute to the interspecific variability in sap flow (O’Brien *et al.*, 2004; Hernandez-Santana *et al.*, 2015). Research typically divides interspecific variation along a fast–slow/growth–survival axes, where pioneer species tend to adopt a ‘live fast and die young’ approach to maximize growth (with high plant water use), while climax or late-successional species adopt a low-risk, slow-growth strategy (with conservative water use) with high survival rates (Rüger *et al.*, 2020; Lai *et al.*, 2021).

In terms of intraspecific variations, these are manifested as radial and azimuthal (between cardinal directions) variability in sap flow and are influenced by growth stage, plant size, stem anatomy (ring-porous versus diffuse-porous species), and individual responses to the microclimate (Fiora and Cescatti, 2006; Berdanier *et al.*, 2016; Berry *et al.*, 2018). Individual plant- and species-level studies are therefore crucial for understanding the responses of co-occurring species belonging to similar or different functional groups to changing environmental conditions, especially in biodiverse tropical montane forests (TMFs; Forrester, 2015; Bretfeld *et al.*, 2018; Basnett and Devy, 2021). Such studies are also crucial to our understanding of the responses of secondary forest communities to the predicted intensifications of hydrological cycles with climate change, especially to extreme precipitation events, seasonal droughts, and reductions in winter precipitation (Donohue *et al.*, 2007; Zeppel *et al.*, 2008, 2011; Kulkarni *et al.*, 2013; Bretfeld *et al.*, 2018). The impact of atmospheric CO_2_ fertilization on plant water use and carbon assimilation rates, and hence on carbon sequestration potential, is another important area of research in TMFs (Bugmann and Bigler, 2011; Eckert *et al.*, 2021).

Yet, ecohydrological studies describing the linkages between micro-environment and ecophysiological traits associated with transpiration and their underlying mechanisms are relatively sparse in TMFs. Most studies have been restricted to the tropical Andes and South-East Asia, and very little research has come from the Himalaya region (Bruijnzeel *et al.*, 2011; Poyatos *et al.*, 2021); such studies that have been carried out have mainly focused on interspecific patterns in leaf water potential with environmental gradients, with most coming from the drier and relatively higher latitudes of Western Himalaya (Zobel *et al.*, 2001; Poudyal *et al.*, 2004; Singh *et al.*, 2006). Direct measurements of whole-tree transpiration are rare, with Ghimire *et al.* (2014) being the first to compare the variability in transpiration rates between needle-leaved Chir pine (*Pinus roxburghii*) and broadleaved oak forests in Central Himalaya using sap-flow probes. Other studies have also highlighted the crucial role of fluctuations in seasonal soil moisture on phenology and growth cycles in broadleaved trees in the Himalaya (Singh *et al.*, 2000; Tewari *et al.*, 2016; Chand *et al.*, 2017). The variability in ecophysiological responses of Himalayan trees to seasonal moisture deficit has also been examined across differing elevations, species ranges, and canopy structures (Zobel *et al.*, 2001; Singh *et al.*, 2006; Tewari *et al.*, 2018). As a result of the long history of human forest use, the majority of broadleaved TMFs in the Himalaya are secondary forests, and they remain understudied (Ramakrishnan and Kushwaha, 2001; Kanade and John, 2018). Secondary TMFs differ significantly from their primary counterparts in that species of different successional stages such pioneer and late-successional co-exist, together with mixed stand structures (sub-canopy and emergent trees) (Ramakrishnan and Kushwaha, 2001; Nogueira *et al.*, 2004). In the Himalayan state of Sikkim in northeast India, secondary TMFs constitute a third of the total forest cover and form a significant carbon sink, and are a source of the springs and streams that provide principal water resources (Wohl *et al.*, 2012; Brown and Lugo, 1990; Daniel *et al.*, 2021; Kumar *et al.*, 2023). The majority of these forests are under protected area management, and understanding their plant water relations is critical to the water and ecological security of the Eastern Himalaya (Kanade and John, 2018). The region is threatened by climate change, with projections indicating a high rate of warming (+5 °C), a significant increase in precipitation (+40%), and a high loss of biodiversity (33%) by the end of the this century (Kulkarni *et al.*, 2013; Krishnan *et al.*, 2019; Dahal *et al.*, 2021). The forests in parts of the Himalaya have already shown trends of browning in response to warming-induced moisture stress in recent decades (Krishnaswamy *et al.*, 2014), making a better understanding of plant water relations a research priority.

To the best of our knowledge, plant water relations and transpiration have not been studied in the wet secondary TMFs of Eastern Himalaya using direct whole-tree methods such as sap flow. Further, the variability in water-use strategies among different functional groups in response to seasonally moist and dry conditions and its impact on plant productivity and the carbon sequestration potential also remains to be explored in these human-influenced biodiverse forest communities (Bretfeld *et al.*, 2018). The current study aims to address this knowledge gap using a response-based approach to understanding the variability in plant water use among co-occurring pioneer and late-successional species in relation to micro-meteorological and environmental factors under a SPAC framework (Mencuccini *et al.*, 2019; Kannenberg *et al.*, 2022). Specifically, this study addresses the following two questions within the context of an East Himalayan broadleaved wet evergreen TMF. (i) How do the patterns of sap flow differ between co-occurring pioneer and late-successional species in a secondary forest? (ii) What are the environmental and ecophysiological drivers of variability in sap flow? We hypothesized that plant water use by the pioneer species would be high and would likely be highly sensitive to environmental extremes, whereas late-successional species would have relatively stable water use patterns. We also predicted that, unlike other TMFs, the ecosystem productivity was more likely to be limited by energy availability than water in the wet East Himalayan eco-climate.

## Materials and methods

### Site description

The forest stand used as the experimental site represents the East Himalayan broadleaved evergreen wet montane forest classification (Sudhakar *et al.*, 2008; Kanade and John, 2018; Bhutia *et al.*, 2019). The site was 2150 m above sea level (masl) with a mean annual precipitation (MAP) of 4650 (±120) mm, making it one of the highest elevation and wettest tropical montane forest (TMF) site to date where direct sap flow-based plant water flow measurements have been carried out (Bruijnzeel *et al.*, 2011; Poyatos *et al.*, 2021). Such TMFs are distinguished from temperate broadleaved evergreen forests (high latitude and low elevation) in terms of being relatively closer to the tropics (20–35°N) and having high elevation (800–2800 masl), a wet/humid climate (MAP 3000–5000 mm), moderate energy conditions (12 °C mean annual temperature), and high biodiversity (Ohsawa, 1993; Tang and Ohsawa, 1999; Kanade and John, 2018). The field study was conducted in a secondary forest stand (27.35°N, 88.56°E) in the Fambong-Lho Wildlife Sanctuary (FWS), Sikkim, India, with annual daily mean temperatures ranging from –2 °C to 24 °C. The hydrologic year at the site is divided into three distinct seasons, as follows. Summer, (March to May), characterized by warm days, cloudy afternoons, significant pre-monsoon precipitation, and high evapotranspiration; monsoon (June to October), with concentrated precipitation, high humidity, and low evapotranspiration; and winter (November to February) with sunny days, cold nights, occasional snowfall, and moderate evapotranspiration (Kumar *et al.*, 2021). The forest is sloped at 10–35°, predominantly facing northeast, and the soil is a well-drained sandy-loam with moderate depth (40–100 cm).

Vegetation data were derived from five vegetation surveys each of 100 × 10 m, which recorded 321 trees of 16 species belonging to 12 families (Bhutia *et al.*, 2019). The composition marked the stand as an early-successional secondary forest with a short-statured canopy dominated by pioneer species such as *Symplocos* and *Eurya* species. Relatively older, taller, and larger-girthed individuals of the *Fagaceae* family, such as *Castanopsis* and *Quercus* species, stood out as emergent trees (Sudhakar *et al.*, 2008; Bhutia *et al.*, 2019; Gurung and Chettri, 2019). Tree density provided a better estimate than basal area for understanding the population structure of pioneer species because of the higher number of small-sized individuals, whereas the late-successional species were larger-sized and fewer in number. The selection of species for sap-flow measurements was based on their representation of the successional stages (pioneer and late-successional), and on selection of dominant species in each of the successional stages according to the tree density and basal area distribution of the stand. *Symplocos racemosa* and *Eurya acuminata* were selected as representing the fast-growing pioneer species community, whilst *Castanopsis hystrix* was selected as the most dominant species belonging to the climax or late-successional stage (Supplementary Fig. S1). Information on rooting depth, phenology, and other ecophysiological characteristics of the three species collated from available literature and personal observations is presented in Table 1. *Symplocos racemosa* was observed to be flowering and fruiting in June–July during the monsoon season, *E. acuminata* flowered in December–January at the peak of the dry season and fruited during February–March, whilst *C. hystrix* flowered in February and fruited in March–April. None of the three species was observed to have any concentrated leaf fall.

**Table 1.** Life-forms and ecophysiological characteristics of the three species

Species	Family	Form	Functional group	Canopy position	Rooting depth	Wood type
*S. racemosa*	*Symplocaceae*	Small-medium trees	Pioneer	In-canopy	Shallow	Diffuse-porous
*E. acuminata*	*Pentaphylacaceae*	Shrubs/small trees	Pioneer	In-canopy	Shallow	Diffuse- porous
*C. hystrix*	*Fagaceae*	Large trees	Late-successional	Emergent	Deep	Semi ring- porous

References: Ohsawa *et al.*, 1986; Suzuki *et al.*, 1991; Sundriyal and Sharma, 1996, 2003; Chettri *et al.*, 2002; Sharma *et al.*, 2011; Li *et al.*, 2013.

### Sap flux measurements

Sapwood thickness was measured from wood cores extracted using an increment borer from each selected tree at the start of the experiment. The cores from *C. hystrix* showed distinct heartwood formation and only the outer xylem (4–5 cm) was observed to be functional. No heartwood was detected in *S. racemosa* and *E. acuminata,* and the entire xylem was assumed to be functional, a characteristic of diffuse-porous species (Berdanier *et al.*, 2016). After installing the probes, wood cores were collected from each tree concurrently (14 December 2013), wrapped in plastic cling film immediately after extraction to avoid moisture loss, and then subsequently processed in the laboratory. Each core was cut into 1-cm long sections to estimate wood density and moisture following the method of Chave (2006). Sap flux measurements were carried out from November 2013 to May 2014 for five trees each of *S. racemosa* and *E. acuminata*, and three trees of *C. hystrix* using the Granier’s thermal dissipation probe (TDP) method (Table 2) (Granier, 1987; Lu *et al.*, 2004). A total sample size of 13 trees and 3–5 replicate trees is consistent with the average number that have been used in previous sap flow studies, and the installation design was optimized to cover maximum xylem variability (Asbjornsen *et al.*, 2011; Guyot *et al.*, 2015). Davis *et al.* (2012) suggested modifications to the lab-built TDP design template to measure sap flow at the top 1 cm of the probe and validated the probes against the commercial Dynamax TDP30 sap flow probe. Our study used lab-built probes based on this modified design template (for further details see Phillips *et al.* 1996; James *et al.* 2002; Davis *et al.* 2012). TDPs the most widely used technique for measuring sap flow and they perform well in cold and low-flow conditions (Lu *et al.*, 2004; Chan and Bowling, 2017; Wang *et al.*, 2023). They are also known to suffer from linear systemic bias and thus are suitable for relative comparisons between species (Peters *et al.*, 2018; Flo *et al.*, 2019). Radial probes of 1–5 cm length were installed at 1 cm incremental depths between 1–5 cm, respectively, from the cambium in one tree for each of the three species (henceforth referred to as ‘radial trees’), in a spiral design. The remaining trees for each species (henceforth referred to as ‘replicate trees’) were fitted with two 2 cm length probes that were installed in the north and south aspects to observe azimuthal variability (Shinohara *et al.*, 2013; Komatsu *et al.*, 2016). A design limitation of using multiple sap-flow probes of different depths in a spiral is the potentially confounding interactions between radial and circumferential variability, which can result in significant biases in the calculation of whole-tree water use, especially in trees with non-uniform xylem growth patterns (James *et al.*, 2002).

**Table 2.** Basic morphological details of the trees measured in the experiment, the number of radial probes installed per species, and number of unique days of sap flow data per species.

Species	No. of trees	DBH (m)	Basal area per tree (m^2^)	Sapwood area per tree (m^2^)	No. of radial probes per tree	No. of unique data days
*S. racemosa*	5	0.21 ± 0.06	0.036 ± 0.02	0.032 ± 0.02	5	49
*E. acuminata*	5	0.19 ± 0.04	0.028 ± 0.01	0.026 ± 0.01	4	87
*C. hystrix*	3	0.34 ± 0.2	0.11 ± 0.12	0.037 ± 0.02	5	99

DBH, diameter at breast height. Means (±SD) are shown.

The probe signals (in mV) were converted to temperature differences (∆*T*, °C) between the heater and reference sensors in a TDP probe at 30 min intervals and stored in a multi-channel data logger (DL2e, Delta-T, UK). The zero-sap flow assumption, at which the temperature difference between the probes is maximum (∆*T*_max_), is critical for Granier’s method and a significant source of error in TDP-based sap-flow estimation. When comparing different approaches for determining ∆*T*_max_, Rabbel *et al.* (2016) showed that daily ∆*T*_max_ has lower variability than studies using moving-window or regression-based approaches, and hence they recommended daily ∆*T*_max_ for research in humid environments without significant water limitations. Consequently, we computed sap flux density (*J*, cm^3^ cm^–2^ h^–1^) following Granier’s empirical equation below, where ∆*T*_max_ was determined daily for each probe:


$$\begin{array}{l} J=\;119\times 10^{-6}\times 3600\times {\left(\frac{\left(\Delta T_{\text{max}}-\;\Delta T\right)}{\Delta T}\right)}^{1.231} \end{array}$$ (1)

The first 2 weeks of data were ignored to avoid errors due to installation wounds (Wiedemann *et al.*, 2016). After quality checks, 114 unique days of sap-flow data between 7 December 2013 and 15 May 2014 were used for the final analysis, and this included 33 d where data were present for all three species (Supplementary Fig. S2; Supplementary Table S1B).

### Environmental measurements

Soil water potential was recorded at 10 cm incremental depths from the surface at 10 min resolution using granular matrix-based (watermark) sensors (Virtual Electronics, Roorkee, India) and converted to volumetric water content using site-specific van Genuchten water retention curve parameters. The curve parameters were developed using the percentage of soil organic matter and organic carbon, particle size distribution (sand, silt, and clay), bulk density, and porosity as input parameters in the Rosetta software (Schaap *et al.*, 2001). The hourly total soil moisture (*S*, in mm) for the topsoil (0–30 cm depth) was computed using the trapezoidal method and smoothed using a three-step moving-average window to fill stray gaps resulting from missing values (Nachabe *et al.*, 2005). In-canopy air temperature and relative humidity were recorded at 10-min resolution (iButton Hygrochrons, Maxim Int., USA). Air temperature (°C), relative humidity (*R*_h_, in %), wind speed (*u*, in m s^–1^), and incoming short-wave radiation (*R*_sol_, in kW m^–2^, henceforth referred to as solar radiation) were recorded with 10 min resolution using an automatic weather station (AWS; Vantage Pro2, Davis Instruments, USA). Vapour pressure deficit (VPD, kPa) was calculated from the hourly air temperature and relative humidity recorded by the Hygrochrons and the AWS and averaged. Precipitation (*P*, in mm h^–1^) was recorded using a tipping-bucket rain gauge (Spectrum Technologies, India) fitted with an odyssey data logger (Dataflow systems Ltd., New Zealand). Data processing, analysis, and visualization were done in the R software (https://www.r-project.org).

### Data analysis

The data analysis consisted of three main steps: scaling from the probes to whole-tree sap flow, assessing sap flow variability within and between species, and linking the environmental drivers of the observed patterns in sap flow. The scaling procedure followed established literature, and relative biases in whole-tree sap flow were estimated. The intraspecies differences in sap flow were investigated within an individual tree and between individuals of the same species along radial (three metrics) and azimuthal (one metric) axes. The interspecific variability was explored between the two co-occurring functional groups, pioneer and late-successional species. Species-wise multiple linear regression models were used to understand the drivers of day and night sap-flow patterns. SPAC interactions were explored using lag regression analysis while accounting for the relative time lags across the different seasons. The observed temporal lag was combined with a generalized least-squares (GLS) regression time-series model to quantify the relative influence of key environmental drivers on sap flow in the three species, including solar radiation and VPD.

#### Scaling from sap-flux density to whole-tree sap flow.

A combination of the zero-averaged technique and weighted mean method was used to estimate the whole-tree sap flow in trees with radial probes (*V*_rad_, in kg h^–1^) (for details, see Hatton *et al.* 1990; Pausch *et al.* 2000). Another estimate of whole-tree sap flow, *V*_rad.2cm_, was calculated in the radial-probe trees using sap flux density at 2 cm depth only (*J*_2cm_) and the total sapwood area (*A*_total_), assuming homogenous radial flow for comparison against *V*_rad_:


$$\begin{array}{l} V_{\text{rad}.2\text{cm}}=\;J_{2\text{cm}}\times A_{\text{total}}\times 10^{-3} \end{array}$$ (2)

*V*_rad.2cm_ showed significant overestimation (15%) in comparison with sap flow estimated using the radial profile *V*_rad_. Previous studies have used simple linear regression models (LRMs) between integrated whole-tree sap flow (*V*_rad_) and sap flow in the outer xylem for trees with only shallow probes (*V*_rad.2cm_) (Paudel *et al.*, 2013; Berdanier *et al.*, 2016). However, given the diel variability in their relationships, we fitted linear regression models without intercepts for each hour of the day for each species (Supplementary Table S2). The slopes (*m*) of the LRMs for each hour were multiplied by the mean sap-flux density between the north- (*J*_north_) and south-facing (*J*_south_) probes and by the total sapwood area (*A*_total_) to estimate whole-tree sap flow in the replicate trees using a linear model (LM) method (*V*_rep.LM_, in kg h^–1^) (Paudel *et al.*, 2013; Berdanier *et al.*, 2016), as follows:


$$\begin{array}{l} V_{\text{rep}.\text{LM}}=\left(\frac{J_{\text{north}}+J_{\text{south}}}{2}\right)\times A_{\text{total}}\times m\times 10^{-3}\; \end{array}$$ (3)

For the subsequent analyses, *V*_rad_ and *V*_rep.LM_ are combined under the term ‘whole-tree sap flow’ and represented by *V* (kg h^–1^).

#### Biases in the estimation of whole-tree sap flow.

The relative biases in whole-tree sap flow were estimated in replicate trees by systematically ignoring radial and azimuthal variability and compared with whole-tree sap flow as estimated by incorporating radial variability (*V*_rep.LM_, Equation 3) (see Shinohara *et al.*, 2013). First, whole-tree sap flow was estimated for replicate trees using the conventional method (CM) without incorporating the radial variability (*V*_rep.CM_, in kg h^–1^) was estimated by multiplying the *A*_total_ by the average *J* determined from the two diametrically opposite 2 cm probes:


$$\begin{array}{l} V_{\text{rep}.\text{CM}}=\left(\frac{J_{\text{north}}+J_{\text{south}}}{2}\right)\times A_{\text{total}}\times 10^{-3} \end{array}$$ (4)

Second, whole-tree sap flow was estimated for replicate trees based on the north-facing side only (*V*_rep.N_, in kg h^–1^) using the sap flux density for the north-facing probes (*J*_north_) and *A*_total_ while ignoring azimuthal variability


$$\begin{array}{l} V_{\text{rep}.\text{N}}=\;J_{\text{north}}\times A_{\text{total}}\times 10^{-3} \end{array}$$ (5)

#### Intraspecific variability in sap flow.

Within a species, the sap-flow variabilities were visualized and quantified as the variabilities in xylem conductivity along the radial and azimuthal profiles. The radial sap flow variability was observed in one tree per species and quantified using three metrics (Delzon *et al.*, 2004; Fiora and Cescatti, 2006), as follows. (i) Daily maximum sap flux density (*J*_max_) was plotted against the depth from the cambium to indicate regions of high sap flux in the sapwood. (ii) The percentage contribution of sapwood annuli at each depth to daily *V* was estimated as a product of the sapwood area of the annulus and the corresponding *J* values. (iii) Radial variability was quantified by means of hourly correction factors (*C*_h_), which were estimated for sunny days for trees with radial probes as the sum of hourly ratios of *J* at different depths (*J*_*i*_) with reference to *J*_2cm_, normalized by the sapwood area of each annulus (*A*_*i*_) over *A*_total_ (Delzon *et al.*, 2004), as follows:


$$\begin{array}{l} C_{\text{h}}=\overset{n}{\underset{i=1}{\sum }}\left(\frac{J_{i}}{J_{2\text{cm}}}\right)\times \left(\frac{A_{i}}{A_{\text{total}}}\right) \end{array}$$ (6)

The azimuthal variability in sap flow was estimated as hourly ratios between *J*_north_ and *J*_south_ (*R*_NS_) and the results were averaged across the replicate trees for each species (Shinohara *et al.*, 2013). The coefficients of variability (CV) were estimated for individual trees and averaged for each species for the key sap flow parameters of sap flux density (*J*), whole-tree sap flow (*V*), nocturnal sap flow (*V*_Night_, see below), and the sap flux ratios (*R*_NS_) (Wang *et al.*, 2018).

#### Interspecific variability in sap flow.

Diel patterns in the sap flow of the three species were assessed for variability across seasons. Our assessment of interspecific variability was focused on the relative differences in sap flow along the radial and azimuthal profiles among the three species. Species-wise multiple linear regression models (MLRMs) were fitted for the day (06.00–18.00 h) and night (18.00–06.00 h) periods to assess the relative roles of the environmental variables, *R*_sol_, VPD, and *S* in driving indices of radial (*C*_h_) and azimuthal (*R*_NS_) variability (Moore *et al.*, 2011; Barbeta *et al.*, 2012). Values of *C*_h_ above and below 1 suggest under- and overestimation, respectively, by the 2 cm probe compared to the rest of the xylem. Similarly, *R*_NS_ values above 1 suggest dominant flow on the north-facing part of the trunk, while values below 1 suggest dominant flow on the south side. Sap flow between 18.00-06.00 h was considered nocturnal (*V*_night_), and its percentage contribution to daily *V* was estimated. *V*_night_ was further divided into evening (18.00-00.00 h, *V*_evening_) and pre-dawn (00.00-06.00 h, *V*_pre-dawn_) components (Forster, 2014). Species-wise MLRMs using *V*_night_ as the response variable and VPD, *S*, and wind speed (*u*) as predictor variables were fitted to quantify the environmental drivers of nocturnal sap flow (Barbeta *et al.*, 2012). In the MLRMs, VPD, *u*, and *S* were normalized by their respective daily means to facilitate the interpretation of the results.

#### Lag analysis between sap flow and SPAC variables.

Exploratory linear regression models yielded poor results for interactions between sap flow and environmental drivers, which were temporally lagged due to phase differences (Bond *et al.*, 2002). Hence, lag correlation analysis was used to compute seasonal changes in the time lag between the environmental drivers and whole-tree sap flow (*V*) (Moore *et al.*, 2011). The time-series for *S* was de-trended to extract the diel signals (*S*_diu_) using the *stlplus* package in R. The hourly lag was computed using the *ccf* function in the *stats* package in R for each day between *R*_s_ versus *V*, VPD versus *V*, and *S*_diu_ versus *V* for each of the three species. The days with significant auto-correlation coefficients (ACF ≥|0.4|) were plotted for the three species. Positive ACF values signify that a high value of the driver variable is followed by a high value of the response variable after the corresponding lag hours. Conversely, negative ACF values signify that a high value of the driver variable is followed by a low value of the response variable. Similarly, positive lag hours indicate that the driver variable leads the response variable and that negative lag hours indicate that the driver variable lags behind the response variable.

#### GLS linear regression models for whole-tree sap flow.

The relative influences of environmental (predictor) variables on the whole-tree sap flow (response) variable were quantified using a combination of a temporal lag and generalized least-squares (GLS) regression models using the *gls* function in the *nlme* package in R with a suitable correlational structure. The GLS regression method estimates the maximum likelihood of the regression coefficients using generalized least-squares and is suitable for analysing time-series data with auto-correlational structures (Pinheiro and Bates, 2006; Krishnaswamy *et al.*, 2012). The GLS model used *R*_s_, VPD, and *S* as predictor variables, and they were normalized by subtracting the daily mean from the time-series and checked for collinearity. The second-order autoregressive moving average (corARMA) was found to be the best-fit correlational structure. The GLS model with corARMA structure was run separately for each tree to capture the interactions at both intra- and interspecific levels. Contiguous subsets longer than 5 d without missing values were tested for the time lag between the driver and response variables using the *ccf* function in R. If any significant lag (ACF ≥|0.4|) was observed, the driver variable was lagged by a suitable range of hours to develop a multiple-lagged time-series starting from zero lag time, and a linear regression model was fitted with the response variable. The procedure was repeated for each of the driver variables, and the lagged time-series with the lowest lag hours (*P* ≤ 0.05) was chosen to develop a reconstituted dataset. The GLS model with corARMA structure was compared against a similar GLS model without any autocorrelation structure using ANOVA. The GLS model with the lowest Akaike information criterion and the highest likelihood ratio and significance was chosen for interpretations. The model performance was assessed using a linear regression model and the Kling–Gupta Efficiency (KGE) score for the observed and predicted values of *V* (Gupta and Kling, 2011).

## Results

### Intraspecific variability in sap flux density and sap flow

#### Radial variability in sap flux density and sap flow.

**Fig. 1.**
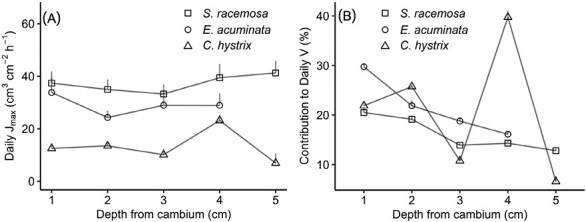
Radial variability in sap flow in the pioneer species *Symplocos racemosa* and *Eurya acuminata* and the late-successional species *Castanopsis hystrix* measured *in situ* in an Eastern Himalayan forest. (A) Mean daily maximum sap-flux density (*J*_max_, cm^3^ cm^–2^ h^–1^) and (B) percentage contribution of the sap flow at different depths to daily whole-tree sap flow (*V*, kg h^–1^). Data are means (+SE), 56 d of measurements including overlapping sample days for *Symplocos racemosa* (Tree 1, *n*=16), *Eurya acuminata* (Tree 1, *n*=12) and *Castanopsis hystrix* (Tree 3, *n*=47).

Radial patterns in *J*_max_ varied considerably across the three species. The pioneer species *S. racemosa* and *E. acuminata* both exhibited concave curves for *J*_max_, with comparable peaks at the outer- and innermost xylem (37.4 ± 15 cm^3^ cm^–2^ h^–1^ and 41.3 ± 23 cm^3^ cm^–2^ h^–1^, respectively; Fig. 1A). Correspondingly, the percentage contribution of each depth to daily *V* declined uniformly from the outermost xylem inwards for these species (Fig. 1B). Conversely, the late-successional species *C. hystrix* showed highest *J*_max_ (23.1 ± 14 cm^3^ cm^–2^ h^–1^) at the inner xylem (4 cm depth from the cambium) and its contribution to daily *V* was disproportionately higher (40%) than the rest of the depths. Following the above patterns, the highest wood moisture and lowest wood density were also observed at 1 cm depth in *S. racemosa* and *E. acuminata*, and at 4 cm depth in *C. hystrix* (Supplementary Fig. S3). Overall, mean hourly *J* increased with wood moisture and declined with wood density for a given sapwood annulus ring, except for *E. acuminata* (Supplementary Fig. S4). The relative contributions of sapwood annuli to whole-tree sap flow were similar for both day and night periods and across the species. The innermost probe (5 cm depth) in *C. hystrix* stopped showing sap flux movement after January, indicating loss of conductivity in the annuli as the inner sapwood transitioned into non-functioning heartwood (Brodersen *et al.*, 2019).

The three species displayed contrasting diel patterns in the variability of the radial correction factor, *C*_h_ (Supplementary Fig. S5A). In *S. racemosa* and *E. acuminata*, the 2 cm probe underestimated sap flow in relation to the rest of the xylem (*C*_h_>1) during the evening-night period (17.00–04.00 h) and overestimated it (*C*_h_<1) during the day (04.00–1700 h). In contrast, in *C. hystrix* the 2-cm probe overestimated sap flow during the night-morning (23.00–09.00 h) and overestimated it during the noon-evening period (10.00–22.00 h). Closer examination indicated that the variability in *C*_h_ was driven by the higher sap-flux density at the outer xylem (1 cm depth) in *S. racemosa* and *E. acuminata* and at the inner xylem (4 cm depth) in *C. hystrix* compared to the rest of the xylem. The results of MLRMs suggested that daytime radial variability was strongly predicted by *R*_sol_ (negative slope) and *S* (positive slope) in *S. racemosa* (*r*^2^=0.43, *P*<0.001), whereas none of the predictors was significant for either *E. acuminata* (*r*^2^=0.01, *P*<0.6) or *C. hystrix* (*r*^2^=0.01, *P*<0.15) (Supplementary Table S3). On the other hand, VPD was a strong negative predictor of night-time *C*_h_ in *E. acuminata* (*r*^2^=0.4, *P*<0.001), but not in *S. racemosa* (*r*^2^=0.02, *P*<0.2) or *C. hystrix* (*r*^2^=0.001, *P*<0.6).

Both *S. racemosa* and *E. acuminata* exhibited consistently high azimuthal sap flux ratios (*R*_NS_) throughout the day (Supplementary Fig. S5B). Interestingly, in *C. hystrix* the northern probes dominated in the morning, with large standard deviations that suggested significant intraspecific variability. The southern probes showed peaks from the afternoon onwards in this species. The MLRMs predicting *R*_NS_ performed poorly across the species (*r*^2^~0.03–0.18; Supplementary Table S4). However, *R*_sol_ (positive slope) was a significant predictor of *R*_NS_ at moderate values of VPD and *S* across the three species (*P*<0.09). Night-time azimuthal variability was best predicted by VPD (*P*<0.06) and *S* (*P*<0.08) in *S. racemosa*, and by VPD in *C. hystrix* (*P*<0.004). *S* was a significant predictor of night-time *R*_NS_ in *E. acuminata* (*P*<0.04).

#### Intraspecific variability in sap flux density and sap flow.

The CV between the three species was very high for all sap-flow parameters (*J*, 255%; *V*, 246%; *V*_Night_, 147%; and *R*_NS_, 236%). The CV in *J* was significantly higher among individuals of *S. racemosa* (282 ± 32%) and *E. acuminata* (267 ± 30%) than *C. hystrix* (179 ± 12%). Similarly, the CV in *V* was significantly higher between individuals of *S. racemosa* (279 ± 49%) and *E. acuminata* (273 ± 22%) than *C. hystrix* (170 ± 5%). The intraspecific variabilities in nocturnal sap flow in the pioneer species *S. racemosa* (155 ± 32%) and *E. acuminata* (140 ± 34%) were almost double that of the late-successional *C. hystrix* (78 ± 3%). Conversely, the CV in azimuthal variability in *C. hystrix* (273 ± 28%) was over twice that of *S. racemosa* (114 ± 56%) and *E. acuminata* (96 ± 60%).

### Interspecific variability in sap flow and environmental drivers

#### Comparisons of sap-flux density and whole-tree sap flow.

The pioneer species *S. racemosa* and *E. acuminata* exhibited double the rates of sap-flux density (*J*) compared to the late-successional species *C. hystrix* (Table 3). The whole-tree sap flow (*V*) was linearly related to the total sapwood area (*r*^2^>0.71, *P*<0.001). The larger girth in *C. hystrix* was offset by a significant proportion of non-conducting heartwood (Table 2), whereas *S. racemosa* and *E. acuminata* showed a significantly higher proportion of conducting sapwood, contributing to high *V* values. Radial bias, due to assumptions of homogenous flow along the xylem, led to overestimations of *V* in the three species. The failure to account for azimuthal variability (azimuthal bias) caused a significant underestimation of *V* in *S. racemosa* and *C. hystrix* but an overestimation in *E. acuminata*.

**Table 3.** Estimates of sap flux density (*J*), whole-tree sap flow (*V*), and percentage biases in the estimation of *V* due to ignoring radial and azimuthal variability.

Species	*J* (cm^3^ cm^–2^ h^–1^)	*V* (kg h^–1^)	Radial bias (%)	Azimuthal bias (%)
*S. racemosa*	28 (2)	2.4 (1.2)	8 (4)	–5 (12)
*E. acuminata*	21 (10)	1.52 (0.8)	1 (3)	5 (12)
*C. hystrix*	13 (5)	2.39 (1.7)	8 (7)	–21 (41)

Data are means (±SD); 114 unique day replicates, tree-wise breakup of number of days is given in Supplementary Table S1B.

#### Diel patterns in sap flow in relation to environmental drivers.

**Fig. 2.**
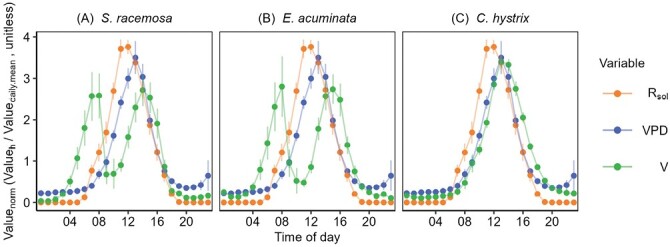
Diel patterns of sap flow (*V*, kg h^–1^) in relation to incoming short-wave radiation (*R*_sol_, kW m^–2^) and vapour pressure deficit (VPD, kPa) in the pioneer species *Symplocos racemosa* and *Eurya acuminata* and the late-successional species *Castanopsis hystrix* measured *in situ* in an Eastern Himalayan forest. The data are normalized by their respective daily means, and are means (±SE), for the 28 concurrent data days which coincided for all species from total days for each species: *Symplocos racemosa* (Tree 1-5, 100 d), *Eurya acuminata* (Tree 1-5, 80 d) and *Castanopsis hystrix* (Tree 1-3, 65 d), a tree-wise breakup of *n* is given in Supplementary Table S1A.

The peak sap flowin *S. racemosa* and *E. acuminata* occurred relatively early in the day (07.00-08.00 h), whereas in *C. hystrix* it peaked around 12.00 h (Fig. 2; Supplementary Table S1A). Both *S. racemosa* and *E. acuminata* showed low sap flow at high values of *R*_sol_ and VPD (Supplementary Fig. S6). The heightened sensitivity to the environmental extremes manifested in bimodal peaks in diel sap flow with significant midday depression of flow across the trees for *S. racemosa* and *E. acuminata*. This depression coincided with peaks in VPD and *R*_sol_ and a trough in *S*. In contrast, *C. hystrix* maintained relatively high *V*at high values of *R*_sol_ and VPD with unimodal peaks, although minor signs of a midday depression in sap flow were seen in two of the three trees.

#### Nocturnal sap flow.

Nocturnal sap flow (*V*_night_) as a fraction of daily *V* was highest in *E. acuminata* (17.2 ± 9%), followed by *C. hystrix* (13.5 ± 5%) and *S. racemosa* (11.3 ± 7%) (Table 4). A significant proportion of *V*_night_ occurred during the pre-dawn period in *S. racemosa* (6.2 ± 7% of daily *V*), whereas evening flux dominated in *C. hystrix* (9.7 ± 5%). The proportion during the evening period (9.5 ± 8%) was marginally higher than during pre-dawn (7.7 ± 7%) in *E. acuminata*. The environmental conditions during the night fluctuated between high wind and high VPD to relatively quieter periods with low VPD. Across the species, high values of *V*_night_ were associated with low VPD (<0.1 kPa), low wind velocity (<0.5 m s^–1^), and moderately saturated soil moisture conditions (Supplementary Fig. S7). The MLR models predicting *V*_night_ concurred with the observations that VPD, soil moisture, and wind acted as limiting variables to nocturnal sap flux (Supplementary Table S5). Soil moisture (negative slope) was a significant predictor of *V*_night_ in *E. acuminata* (*r*^2^=0.12, *P*<0.05) and VPD (negative slope) was a significant predictor for *C. hystrix* (*r*^2^=0.13, *P*<0.009). None of the predictors were significant for *S. racemosa* (*r*^2^=0.02, *P*>0.5). Both evening and pre-dawn sap flow were independent of tree size in the three species.

**Table 4.** Percentage contributions of day, whole-night, evening, and pre-dawn sap flows to total daily sap flow in the three species.

Species	Day (06.00-17.00 h)	Night (18.00-05.00 h)	Evening (18.00-23.00 h)	Pre-dawn (00.00-05.00 h)
*S. racemosa*	88.7 (7)	11.3 (7)	5.1 (5)	6.2 (7)
*E. acuminata*	82.8 (9)	17.2 (9)	9.5 (8)	7.7 (7)
*C. hystrix*	86.5 (5)	13.5 (5)	9.7 (5)	3.8 (4)

Data are means (SD); 114 unique day replicates, tree-wise breakup of number of days is given in Supplementary Table S1B.

#### Seasonal changes in diel patterns of environment, sap flow, and transpiration.

**Fig. 3.**
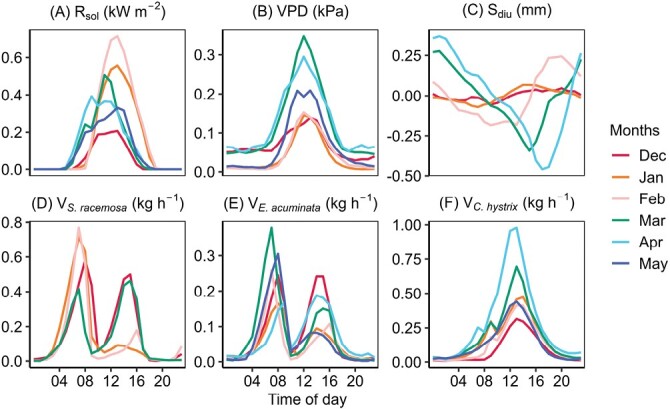
Seasonal changes in diel patterns of environmental variables and whole-tree sap flow in the pioneer species *Symplocos racemosa* and *Eurya acuminata* and the late-successional species *Castanopsis hystrix* measured *in situ* in an Eastern Himalayan forest. (A) Incoming short-wave radiation (*R*_sol_), (B) vapour pressure deficit (VPD), and (C) the diel component of soil moisture (*S*_diu_). (D–F) The whole-tree sap-flow rates (*V*) of (D) *S. racemosa*, (E) *E. acuminata*, and (F) *C. hystrix*. Each line represents the monthly mean value. The sap-flow rates are based on 114 concurrent data days for the three species: *Symplocos racemosa* (Tree 1-5, 164 d), *Eurya acuminata* (Tree 1-5, 164 d) and *Castanopsis hystrix* (Tree 1-3, 206 d), a tree-wise breakup of *n* is given in Supplementary Table S1B.

Changes in diel patterns of the SPAC variables as the growing season progressed from winter to summer are shown in Fig. 3. The winter months of December–February marked the dry season with sunny but cold days, sub-zero night temperatures, low VPD, and declining soil moisture. In March, rains replenished the soil moisture reserves and promoted leaf flush. The summer months of March–May saw increased day length, abundant moisture, warmer temperatures, and higher VPD. Consequently, *V* also increased from winter to summer, but with considerable variation across the three species (Fig. 3D–F). Both *S. racemosa* and *E. acuminata* exhibited peak sap flows in December and March, whereas *C. hystrix* had peaks in March and April. The intraspecific variability in sap flow also followed similar patterns. Radial variability in sap flux (differences in *J*_max_ between inner and outer xylem) increased in the dry season (December) for *S. racemosa*, whereas *C. hystrix* exhibited the highest variability in March and April (Supplementary Fig. S8). The fractional contribution of the outer xylem to daily *V* increased from December to March for *E. acuminata* and *C. hystrix*, whereas there were only marginal differences observed in *S. racemosa* (Supplementary Fig. S9). Sap flux ratios (*R*_NS_), the metric for azimuthal variability, also increased from winter to summer for *S. racemosa*, whereas in *C. hystrix* it was highest in January (Supplementary Fig. S10). The peak sap flow values increased as the growing season progressed, except for *S. racemosa*, and the daily timing of peak *V* remained consistent across the species (Fig. 3D–F). The bimodal peaks in diel sap flow were consistent for *S. racemosa* and *E. acuminata* across the seasons, with higher rates in the morning than in the afternoon. As noted above, during the dry winter, a mild midday depression in sap flow was observed in two out of three trees of *C. hystrix*, resulting in a shift to unimodal peaks being observed from March onwards. Weak declining trends in *V*_night_ were observed from the dry to the wet season in *S. racemosa* and *C. hystrix*Supplementary Fig. S11. The diel peaks in soil moisture (*S*_diu_) shifted from the afternoon to the evening and had increasing amplitude from winter to summer (Fig. 3C).

### Environmental controls on transpiration

#### Lag analysis between transpiration and SPAC variables.

**Fig. 4.**
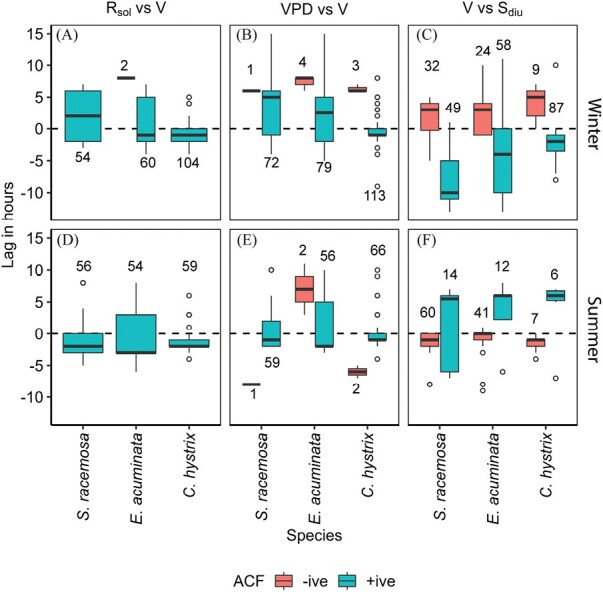
Boxplots showing seasonal shifts in daily lag hours at maximum values of auto-correlation coefficients (ACFs) between environmental variables and whole-tree sap-flow rates of the pioneer species *Symplocos racemosa* and *Eurya acuminata* and the late-successional species *Castanopsis hystrix* measured *in situ* in an Eastern Himalayan forest. (A–C) Values recorded in winter and (D–F) values recorded in summer. (A, D) Incoming short-wave radiation (*R*_sol_, kW m^–2^) versus whole-tree sap-flow rate (*V*, kg h^–1^), (B, E) vapour pressure deficit (VPD, kPa) versus *V*, and (C, F) *V* versus the diel component of soil moisture (*S*_diu_, mm). The colour of the boxplot represents the direction of the ACF values, as indicated, and the values adjacent to the boxes are the number of data points.

*V* consistently lagged behind solar radiation (*R*_sol_) in all three species by an average of 1–2 h with positive correlations in and summer, with the lowest variability in *C. hystrix* (Fig. 4A, D, *R*_sol_ versus *V*). However, in winter, *V* preceded *R*_sol_*in S. racemosa* and *E. acuminata* by an average of 1-2 h with positive correlations. In *S. racemosa* and *E. acuminata*, VPD lagged *V* in both seasons with positive correlations. In contrast, VPD led *V* in both seasons in *C. hystrix* (Fig. 4B, E, VPD versus *V*). On closer inspection, the days when *V* preceded *R*_sol_ and VPD with strong positive correlations were marked by significant pre-dawn flux, and the frequency of such days was significantly higher in *S. racemosa* and during the dry winter months of January and February (Fig. 2). Interactions between *V* and *S*_diu_ were more complicated (Fig. 4C, F, *V* versus *S*_diu_). On most days in winter (75% of total days), across the three species *V* led *S*_diu_ with an average lag of 2.2–7.5 h and positive correlations, while on the remaining days there were negative correlations and *V* lagged behind *S*_diu_ by an average lag of 2.2–4.1 h. However, the patterns were reversed from March onwards with the advent of rains, and on most days in summer (77% of total), *S*_diu_ led *V* by an average of 1.1-1.6 h with negative correlations in all three species, while on the remaining days *S*_diu_ lagged behind *V* by an average of 0.7–4 h and there were positive correlations. We also observed that the lag between transpiration and soil moisture was also positively correlated to tree size, with the larger-sized trees (>0.15 m DBH) experienced a smaller lag between transpiration and soil moisture in the winter in *S. racemosa* and *E. acuminata*, and in the summer in *C. hystrix*, but the results were not significant.

#### GLS linear regression model for whole-tree sap flow.

The sap-flow modelling exercise was carried out for contiguous data periods (without missing values; Supplementary Fig. S2E–G) across winter and summer for the instrumented trees of *S. racemosa* (five periods, 8 ± 2 d), *E. acuminata* (four periods, 10 ± 5 d), and *C. hystrix* (10 periods, 10 ± 4 d) (Table 5). The GLS model with corARMA structure had significantly lower Akaike information criterion than the GLS model without any correlational structure. In summer, VPD (positive slope) emerged as a significant predictor for the three species of whole-tree sap flow (*V*) at average values of *R*_sol_ and soil moisture (*S*). In winter, *R*_sol_ was a co-driver of *V* for *E. acuminata*, and *C. hystrix* with positive coefficients, while *S* was a significant predictor of *V* in one of the trees of *C. hystrix* with a positive coefficient. Interestingly, at least one replicate tree from each species showed a negative *R*_sol_ coefficients in either the winter and/or summer, albeit with low significance (*P*>0.1), indicating the occasionally limiting role played by *R*_sol_. *S* was not a significant predictor of *V*; however, it is important to note that soil moisture always remained well above the permanent wilting point of the site. The relative strength of interactions between *V* and the environmental drivers varied between individual trees of the same species and across the seasons.

**Table 5.** Results from generalized least-squares linear regression models with corARMA correlational structure for predictor variables versus whole-tree sap flow.

Species	Tree	Season	No. of data days	Predictor variables	Coefficients	SE	Student’s *t*	*P*
*S. racemosa*	Tree 2	Summer	9	(Intercept)	0.369	0.09	4.092	<0.001***
				*S*	0.014	0.067	0.204	0.839
				*R*_sol_	–0.337	0.561	–0.601	0.548
				**VPD**	**1.768**	**0.883**	**2.002**	0.047*
	Tree 3	Winter	5	(Intercept)	1.164	0.447	2.605	0.01**
				*S*	–0.159	3.502	–0.045	0.964
				*R*_sol_	–0.41	1.715	–0.239	0.812
				VPD	6.832	7.653	0.893	0.374
		Summer	8	(Intercept)	0.564	0.058	9.703	<0.001***
				*S*	0.041	0.041	1.013	0.312
				*R*_sol_	0.413	0.362	1.142	0.255
				**VPD**	**3.481**	**0.554**	**6.283**	<0.001***
	Tree 4	Summer	8	(Intercept)	0.255	0.052	4.905	<0.001***
				*S*	0.016	0.033	0.506	0.614
				*R*_sol_	0.014	0.223	0.061	0.952
				**VPD**	**1.199**	**0.358**	**3.348**	<0.001***
	Tree 5	Summer	8	(Intercept)	0.14	0.023	5.954	<0.001***
				*S*	0.009	0.016	0.586	0.559
				*R*_sol_	0.148	0.122	1.217	0.225
				**VPD**	**0.619**	**0.189**	**3.282**	<0.001***
*E. acuminata*	Tree 2	Summer	8	(Intercept)	0.146	0.025	5.901	<0.001***
				*S*	0.004	0.018	0.216	0.829
				*R*_sol_	0.165	0.134	1.235	0.219
				**VPD**	**0.643**	**0.204**	**3.16**	0.002**
	Tree 3	Summer	9	(Intercept)	0.186	0.057	3.269	<0.001***
				*S*	0.001	0.043	0.016	0.987
				*R*_sol_	–0.052	0.36	–0.146	0.884
				VPD	0.297	0.562	0.528	0.598
	Tree 4	Winter	16	(Intercept)	0.342	0.045	7.606	<0.001***
				*S*	0.169	0.091	1.86	0.064
				***R***_**sol**_	**0.56**	**0.082**	**6.793**	<0.001***
				**VPD**	**3.128**	**0.268**	**11.669**	<0.001***
		Summer	5	(Intercept)	0.869	0.474	1.836	0.069
				*S*	–0.01	0.317	–0.032	0.974
				*R*_s_	–1.903	1.329	1.432	0.155
				VPD	0.901	2.984	0.302	0.763
*C. hystrix*	Tree 1	Winter	9	(Intercept)	0.48	0.128	3.745	<0.001***
				***S***	**0.716**	**0.3**	**2.384**	0.018*
				***R***_**sol**_	**3.18**	**0.347**	**9.165**	<0.001***
				**VPD**	**4.993**	**0.842**	**5.927**	<0.001***
			16	(Intercept)	0.917	0.121	7.55	<0.001***
				*S*	–0.395	0.455	–0.868	0.386
				***R***_**sol**_	**1.59**	**0.365**	**4.358**	<0.001***
				**VPD**	**11.087**	**1.298**	**8.541**	<0.001***
		Summer	13	(Intercept)	1.518	0.179	8.489	<0.001***
				*S*	0.1	0.063	1.591	0.113
				***R***_**sol**_	**2.443**	**0.417**	**5.857**	<0.001***
				**VPD**	**8.324**	**0.875**	**9.517**	<0.001***
			10	(Intercept)	1.994	0.376	5.299	<0.001***
				*S*	0.105	0.164	0.639	0.523
				*R*_sol_	0.3	0.44	0.684	0.495
				**VPD**	**7.807**	**1.191**	**6.555**	<0.001***
	Tree 2	Winter	9	(Intercept)	1.484	0.341	4.346	<0.001***
				*S*	0.75	1.359	0.552	0.581
				*R*_sol_	2.1	1.411	1.488	0.138
				**VPD**	**14.652**	**3.383**	**4.331**	<0.001***
			16	(Intercept)	2.006	0.09	22.228	<0.001***
				*S*	–0.076	0.503	–0.152	0.879
				***R***_**sol**_	**5.618**	**0.364**	**15.417**	<0.001***
				**VPD**	**6.347**	**1.381**	**4.596**	<0.001***
		Summer	7	(Intercept)	3.072	0.795	3.863	<0.001***
				*S*	0.174	0.706	0.246	0.806
				***R***_**sol**_	**–9.826**	**3.332**	**–2.949**	0.004**
				**VPD**	**21.676**	**6.44**	**3.366**	0.001***
	Tree 3	Winter	5	(Intercept)	0.865	0.362	2.392	0.018*
				*S*	0.559	1.456	0.384	0.702
				*R*_sol_	0.211	1.398	0.151	0.88
				VPD	4.181	3.218	1.299	0.196
			9	(Intercept)	0.725	0.072	10.015	<0.001***
				*S*	–0.065	0.18	–0.358	0.72
				***R***_**sol**_	**1.447**	**0.125**	**11.541**	<0.001***
				**VPD**	**5.374**	**0.408**	**13.171**	<0.001***
		Summer	6	(Intercept)	1.248	0.312	4.007	<0.001***
				*S*	–0.141	0.199	–0.71	0.479
				***R***_**sol**_	**3.879**	**0.862**	**4.5**	<0.001***
				VPD	0.52	1.757	0.296	0.768

Predictor variables: *S*, soil moisture (mm); *R*_sol_, incoming short-wave radiation (kW m^–2^); VPD, vapour pressure deficit (kPa); significant values are highlighted in bold (*P<0.05, **P<0.01, ***P<0.001).

In *S. racemosa*, VPD was the only significant predictor of *V* in all the trees in summer (Table 5). In *E. acuminata,* VPD was the only significant predictor of *V* in one of the replicate trees in summer, while in another, VPD and *R*_sol_ were significant co-predictors in winter. In *C. hystrix*, VPD and *R*_sol_ were significant co-predictors in six out of ten periods across both seasons with positive coefficients, except for one period in Tree 3 in summer where *R*_sol_ showed a negative coefficient. The GLS model performed relatively better for *S. racemosa* (*r*^2^=0.15, *P*<0.001, KGE=0.12) than *E. acuminata* (*r*^2^=0.08, *P*<0.001, KGE=0.02) with improved concurrence between predicted and observed *V*. In both these species, the predicted *V* failed to replicate the bi-modal patterns in the observed *V* (Supplementary Fig. S12A, B). However, the model performance was significantly better for *C. hystrix* (*r*^2^=0.41, *P*<0.001, KGE=0.43), with the predicted *V* during the daytime matching well with the unimodal pattern (Supplementary Fig. 12C). However, the predicted *V* had sap flow in the night hours for all three species, which was absent in the observed *V*.

## Discussion

### Influence of secondary forest structure on sap flow variability in co-occurring species

The root–trunk–leaf connectivity in the xylem is a fascinating area of research under the soil–plant–atmosphere–continuum (SPAC), and it is largely dominated by the century-old cohesion–tension theory and piped model of water transport (Kim *et al.*, 2014). The unit-pipe model postulates direct connections between the roots and lateral branches (known as sectoral flow) and is a common feature of ring-porous species. Conversely, diffuse-porous species exhibit considerable lateral connectivity (known as integrated flow), where multiple parts of the crown are supported by a cross-section of the xylem. Interestingly, pruning experiments show that, within the same individual, the outer xylem can exhibit sectoral connectivity, while the inner xylem can show an integrated flow pattern (Čermák and Nadezhdina, 2011; Dong *et al.*, 2019). In this study, the observed Gaussian radial profile with increasing sap-flux density in the inner xylem in *C. hystrix* (Fig. 1), a ring-porous species, suggests sectoral connectivity, which is also typical of isolated trees with an extended crown (Fiora and Cescatti, 2006). The Gaussian radial profile is ascribed to higher light availability to the older portions of the crown around the periphery, which are anatomically connected to the inner xylem. Conversely, in trees forming a continuous canopy, such as *S. racemosa* and *E. acuminata* (both diffuse-porous species), the activity of the lateral foliage is suppressed by shading, and only the top portion of the crown receives adequate light, inducing higher sap-flux density in the outer xylem. This was supported by the overestimation of radial correction factors (*C*_h_<1) that we observed in the outer xylem of the in-canopy pioneer species *S. racemosa* and *E. acuminata*, and underestimation (*C*_h_>1) in the outer xylem in the late-successional emergent trees of *C. hystrix* (Supplementary Fig. S5A). Delzon *et al.* (2004) observed that the radial correction factors were independent of meteorological factors and better represented the connectivity between xylem and crown–water use.

Landscape characteristics such as slope and aspect influence the diel changes in the angle and intensity of incident light, potentially inducing azimuthal variability in sap flow (Kumagai *et al.*, 2007; Berry *et al.*, 2016). Studies have shown greater connectivity between lateral branches (and leaves) and fine-root development within the same aspect of a given tree (Čermák and Nadezhdina, 2011; Dong *et al.*, 2019). Along the same lines, we observed that fully exposed crowns of *C. hystrix* exhibited shifting peaks in sap flow from north- to south-facing xylem following the north-east–south-west trajectory of sunlight, whereas the in-canopy *S. racemosa* and *E. acuminata* maintained dominant sap flow in the north-facing xylem (Supplementary Fig. 5B). Interestingly, the CV in azimuthal variability was almost double in the emergent trees of *C. hystrix* compared to the in-canopy pioneer species *S. racemosa* and *E. acuminata*. Daytime radial and azimuthal variability in the three species was attributable to the interaction between access to solar radiation and soil moisture, whereas night-time variability was negatively correlated with VPD (Supplementary Tables S2, S3); Fiora and Cescatti, 2006; Shinohara *et al.*, 2013). The biases in estimating *V* due to ignoring azimuthal and radial variability were significant and comparable to existing literature (Fiora and Cescatti, 2006; Shinohara *et al.*, 2013).

### Differential water-use strategies among pioneers and late-successional species

Functional groups play a major role in driving interspecific variability in transpiration (Nogueira *et al.*, 2004; Motzer *et al.*, 2005). Our observations of high sap-flux density (*J*) in the pioneer species *S. racemosa* and *E. acuminata* (Fig. 1) align well with a previous study of co-occurring tree species in a semi-arid region of China that showed high transpiration rates in pioneer species, especially in the growing season (Lyu *et al.*, 2020), and with a study from wet Brazilian rain forests, where Nogueira *et al.* (2004) reported pioneer species showing higher intrinsic water use efficiency (the ratio of net photosynthetic CO_2_ assimilation to stomatal conductance) and instantaneous transpiration efficiency (ratio of photosynthesis rate to transpiration) than late-successional species, while maintaining high sap-flux rates. Similarly, other studies have reported higher leaf-level hydraulic conductance and photosynthetic capacity in pioneer species than in late-successional species from South American tropical montane forests (TMFs; Sobrado, 2003; dos Santos *et al.*, 2019) and from Central Panama (Bretfeld *et al.*, 2018).

Like true pioneers, *S. racemosa* and *E. acuminata* are the first tree species to occupy any disturbance-related openings, but they also occur as sub-canopy associates to late-successional *Fagaceae* species in old-growth TMFs in the Sikkim region where our study was located, indicating long-term recruitment of pioneer species. Expanding the conventional segregation of species along the fast–slow/growth–survival axes, Rüger *et al.* (2020) suggested a third axis of stature–recruitment trade-offs that distinguishes long-lived pioneers (fast-growing, longer-lived, attaining tall canopy stature but low recruitment) from short-lived breeders (fast-growing, short-statured, low survival but a high number of offspring). In a meta-analysis of over 5000 species across 13 long-term monitoring plots, Kambach *et al.* (2022) showed that the fast–slow continuum and stature–recruitment are independent life-history strategies that shape tropical secondary forests globally. Although long-term studies are lacking from the landscape of our study, *S. racemosa* and *E. acuminata* can be characterized as long-lived pioneers (LLPs) owing to their ability to grow fast during the initial period after disturbance whilst also being able to adapt and live longer as the forest matures. LLPs are critical secondary forest species and dominate the intermediate successional stages in terms of carbon sequestration and water use (Rüger *et al.*, 2020; Lai *et al.*, 2021).

The pioneer species *S. racemosa* and *E. acuminata* also displayed increased radial and azimuthal variability in the summer (Supplementary Figs S8, S10) and consistent midday depression in *J* (Fig. 2), an indicator of efficient stomatal control over plant water use. Our GLS modelling results showed that at least one tree from each of the three species displayed a limiting effect of solar radiation (*R*_s_) on *V* (i.e. negative coefficients; Table 5). The heightened sensitivity to environmental extremes in pioneer species can be attributed to their canopy position, which exposes them to strong levels of sunlight and VPD, causing midday stomatal closure (Huc *et al.*, 1994; Franco and Lüttge, 2002; Chiariello *et al.*, 2006). It is also attributable to their shallow-rootedness and sensitivity to diel and seasonal soil moisture fluctuations (Pavlis and Jeník, 2000), although the moisture observed in the topsoil (0–30 cm depth) in our study never went below the permanent wilting point. Bretfeld *et al.* (2018) quantified the impact of El Niño-induced drought years on sap flow in 76 species across different successional stages (8–80-year-old forests) in Central Panama and observed that the limiting role of seasonal soil moisture stress was stronger in pioneer species than in late-successional species. Similar reports from Central Himalayan species belonging to the *Fagaceae*, including *Castanopsis indica*, *Quercus semicarpifolia*, *Q. leucotrichophora*, and sub-canopy *Rhododendron arboreum*, have shown that seasonal soil moisture stress can induce a midday slump in leaf water potential (Zobel *et al.*, 2001; Poudyal *et al.*, 2004; Tewari *et al.*, 2018). The absence of significant midday depression in deep-rooted species such as *C. hystrix* at the peak of the dry season and strong diel cycles in soil moisture provide evidence of vegetation accessing moisture from deeper soil layers (Tanaka *et al.*, 2004; Allen, 2014; Kumar *et al.*, 2022, Preprint).

Our observations of 1–2 h of lag between *R*_sol_ and VPD and *V* were seasonally consistent (Fig. 4) and comparable with a previous report from a TMF in Costa Rica (Moore *et al.*, 2018) and marginally higher than a reports from a TMF in Tibet (~1 h lag) (Wang *et al.*, 2017). Since these are stem-based measurements, the lags could be confounded by delays between stem sap flow and canopy transpiration due to hydraulic capacitance and resistance, although such lags are usually ≤1 h (Phillips *et al.*, 1997; Burgess and Dawson, 2008). The longer lags (>1 h) that we observed might also represent delays induced by low morning temperatures in winter and by leaf wetness in the summer due to night rains or dew (Aparecido *et al.*, 2016; Bretfeld *et al.*, 2018; Moore *et al.*, 2018). Although rains in summer usually start around noon, significant rainfall also occurred in the pre-dawn period (Kumar *et al.*, 2021). Hence, incorporating leaf wetness as a variable is recommended for future studies from wet TMFs such as those of Eastern Himalaya that experiences significant summer rains.

### Plant water relations in wet and high-elevation Eastern Himalayan TMFs

#### High sap-flow rates in wet TMF climates.

The observed values of *J* in the three species in our study were significantly higher than their respective conspecifics from other TMFs. Thus, the *J* in *C. hystrix* at our site was found to be three times higher than conspecific *C. tribuloides* from the relatively drier Central Himalaya (MAP=1331 mm; Ghimire *et al.*, 2014). Similarly, the values of *J* that we recorded for *S. racemosa* and *E. acuminata* were 6–9 times higher than conspecifics from drier environments, namely *S. ramosissma* in China (MAP ~2000 mm) and *S. kuroki* and *E. japonica* in Japan (MAP ~1700 mm) (Chiu *et al.*, 2016; Zhang *et al.*, 2018). Observations of higher sap-flux density and sap flow in pioneer species than in late-successional species have been previously reported from tropical lowland secondary forests of Costa Rica (40 masl), and high-elevation TMFs in Panama (1800 masl) and the Ecuadorian Andes (~2000 masl), and are commonly attributed to higher water movement potential in pioneer species (Motzer *et al.*, 2005; McCulloh *et al.*, 2011). The potential role of soil mycorrhizae and root hair densities in facilitating the high transpiration rates observed in these forests is another recommended area for future research (Carminati *et al.*, 2017).

#### Nocturnal sap flow in Himalayan tree species.

The nocturnal sap flows that we observed constitute one of the first records for the pioneer species *S. racemosa* and *E. acuminata* globally and for *C. hystrix* from TMFs. For *C. hystrix*, previous reports from higher latitudes in China have been significantly lower (4% of daily *V*) than our observations (Chen *et al.*, 2018; Wang *et al.*, 2018). In contrast to a study by Wang *et al.* (2018), the observed intraspecific variability in nocturnal sap flow was less than the interspecific variation in late-successional *C. hystrix* However, in agreement with Kangur *et al.* (2021), higher intraspecific variability in nocturnal sap flow was seen in the pioneer species *S. racemosa* and *E. acuminata*, indicative of a broader range of responses to similar environmental stressors. According to the literature, the ecophysiological mechanisms driving nocturnal sap flux can be broadly categorized into refilling of the stem, facilitating night respiration, and endogenous stomatal controls. In our study, the majority of the nocturnal sap flux occurred in the evening (Table 3) and can be attributed to the capacitive refilling of the stem to capacity (Wang *et al.*, 2018; Kamakura *et al.*, 2021). In similar reports from broadleaved evergreen species in East Asia, nocturnal sap flux has been attributed to stem recharge and it has shown low correlations with environmental variables such as VPD (Chen *et al.*, 2018; Wang *et al.*, 2018; Zhang *et al.*, 2020). Nocturnal movement of water to restore hydraulic equilibrium between roots, shoots, and leaves is a known feature of large trees (although not restricted to them), and in our study both evening and pre-dawn sap flux were independent of tree size (Zeppel *et al.*, 2011; Siddiq and Cao, 2018).

Interestingly, Chen *et al.* (2018) also reported significantly higher nocturnal sap flux in tree species with the capacity for corticular photosynthesis (*C. hystrix* being one of the common study species), indicating a role in facilitating respiration. They also highlighted the role of nocturnal sap flux in oxygen transport to the internal xylem during nocturnal respiration, a possible mechanism to alleviate hypoxia-like conditions created within internal photosynthesising xylem tissue during respiration. Marks and Lechowicz (2007) have further shown that nocturnal sap flux is closely linked to leaf nitrogen concentrations and higher rates of extension growth in fast-growing, shade-intolerant pioneer species and they attributed this to nocturnal respiration, which facilitates carbohydrate translocation and other processes associated with the growth of meristems in roots and shoots.

Lastly, the role of endogenous (circadian-driven) stomatal control in regulating nocturnal sap flux under conditions of stable VPD, low wind, and moderate soil moisture merits further investigations (Resco de Dios *et al.*, 2013; De Dios *et al.*, 2015). Under controlled conditions, Chu *et al.* (2009) demonstrated a significant positive correlation between wind speed and nocturnal sap flux up to a threshold when stomatal closure is assumed to limit transpiration. Similar to our observations, in a garden experiment involving temperate broadleaved species, Kangur *et al.* (2021) found significant nocturnal (pre-dawn) stomatal conductance in anisohydric pioneer species in comparison to isohydric late-successional species under hydrated conditions, and they attributed this to contrasting life-history traits dictating stomatal control. Kumar *et al.* (2021) observed a significant diel cycle in summer precipitation, starting from around noon and leaving a short 5–6 h window conducive to plant productivity and transpiration. Pre-dawn flux is an uncommon observation from the Himalaya and, as Barbeta *et al.* (2012) observed in the Mediterranean, could potentially be an adaptation to ensure hydraulic saturation in the stem, to avoid leaf-level moisture leakage, and to maximize photosynthetic CO_2_ uptake at the start of the day. The prevalence of the phenomenon across both pioneer and primary tree species suggests that these traits might indeed be conserved as an adaptation and they merit further investigation (Kangur *et al.*, 2021).

### Energy versus moisture limitations on sap flow in Eastern Himalaya

Our study site experienced strong seasonal fluctuations in environmental conditions, with moisture- and energy-limited winters. The resulting seasonal variability in the interactive roles of sunlight and VPD as drivers of sap flow have been previously reported for TMFs (Moore *et al.*, 2018). We observed that VPD was a stronger driver of sap flow than *R*_sol_ and *S* in both summer and winter, independent of functional groups (Fig. 4), which is in agreement with reports from Central Himalaya (Ghimire *et al.*, 2014) and Central Panama (Bretfeld *et al.*, 2018). However, during periods of sufficient soil moisture but limited energy availability in winter, sap flow was also driven by *R*_sol_ (Table 5), similar to observations from a TMF in Southern Andes (Motzer *et al.*, 2010) and from the Alps (Fiora and Cescatti, 2006). As discussed above, we also found evidence of photosensitivity in the study species through the inhibitive impact of *R*_sol_ on sap flow, similar to reports from Brazilian TMFs (dos Santos *et al.*, 2019). Soil moisture was both a limiting variable for pioneer species, as seen by the positive lags and negative autocorrelation coefficients, and a resource depleted by vegetation water use (Fig. 4C, F), something that particularly applies for the deep-rooted and large-sized individuals of *Fagaceae* (late-successional species) (Kumar *et al.*, 2022, Preprint). Our results correspond to similar observations from Central Panama where older and large-sized late-successional species continue transpiring unperturbed even under drought conditions, while younger pioneer species reduce their water use (Bretfeld *et al.*, 2018). In this respect, the effects of sapwood area (as a parameter of conducting capacity of the tree) and tree size (representing the age and form of the tree) on the ability of different species to withdraw water from different soil layers and possibly groundwater, becomes of particular interest. The linear increase in whole-tree sap flow with sapwood area is likely to plateau for mature individuals, even under saturated soil moisture conditions, due to limits imposed by stomatal conductance (Gao *et al.*, 2015).

Our observations provide empirical evidence for the theory that the East Himalayan forests are energy-limited under abundant moisture conditions (Sebastian *et al.*, 2019), although VPD remains the primary driver of sap flow independent of successional stages, and soil moisture plays a limiting role for pioneer species only (Bretfeld *et al.*, 2018). Under such conditions, together with VPD, solar radiation could be a significant driver of non-monsoon vegetation productivity, as observed by Sebastian *et al.* (2019) using remotely-sensed enhanced vegetation index. The role of solar radiation is further accentuated in uneven-aged secondary forests in the Himalaya, where the positioning of the trees in the canopy determines the spatial availability of solar radiation to different parts of the crown (Fiora and Cescatti, 2006; Küppers *et al.*, 2008; Zhang *et al.*, 2019). The performance of our GLS regression model was affected by the inherent variability in sap flow, with complex phenomena such as midday depression and pre-dawn sap flux dominating the diel patterns of the pioneer species at the site.

### Implications of changing water availability on carbon sequestration in Himalayan TMFs

Climate change studies from Eastern Himalaya have highlighted an increase in mean annual temperature of 0.02 °C year^–1^ with an estimate for daily mean temperature to increase by 1.8–4 °C by the end of the current century and precipitation to increase by 30–40% (Singh *et al.*, 2011; Shrestha *et al.*, 2012). Kulkarni *et al.* (2013) used a high-resolution PRECIS model to project an increase in temperature of up to 5°C and a 40% increase in precipitation by the year 2098 in Eastern Himalaya. In line with these regional trends, Dimri *et al.* (2018) used the Asian Precipitation-Highly Resolved Observational Data Integration Towards Evaluation of Water Resources (APHRODITE), a gauge-interpolated gridded precipitation product, to confirm increasing trends in pre-monsoonal (March–May) and monsoonal (June–September) precipitation in the Sikkim Himalaya region for 1970–2005. Sharma and Goyal (2020) used four global circulation models and an IMD 0.25° gridded precipitation dataset to project rises in maximum daily temperature of 0.4–1.1 °C and 0.5–1.7 °C and increases in mean annual precipitation of 220–380 mm and 150–760 mm under Representative Concentration Pathways (RCPs) 4.5 and RCP 8.5, respectively, for our study region for 2011–2100 (covered under SB6 basin in the paper). The projected rise in temperature is likely to increase both day and night-time VPD and lead to higher sap-flow rates, which we have already observed to be among the highest from tropical montane or lowland forests. The high sap-flow rates may be further increased by the predicted increases in summer and monsoon precipitation (Mcvicar *et al.*, 2010). In winter, the reduced precipitation (low moisture availability) and increased warming (high VPD) may create seasonal drought conditions and induce water stress, especially among pioneer species.

Pioneer species are reported to have relatively high photosynthetic plasticity and leaf hydraulic conductivity that permit high sap-flux rates, while also being vulnerable to xylem embolism due to lower control over water use (Sobrado, 2003; Nogueira *et al.*, 2004; dos Santos *et al.*, 2019). Conversely, despite lower photosynthetic plasticity and lower hydraulic conductance, late-successional species are seemingly unaffected by seasonal drought conditions in winter due to better control of water use through stomatal closure and access to deeper moisture reserves (Sobrado, 2003; Tanaka *et al.*, 2004; Allen, 2014). In addition, higher VPD is also likely to negatively affect nocturnal transpiration, which might in turn affect the ability of these tree species to utilize the available moisture efficiently. However, the net effect could be easily offset by the potential increase in daytime transpiration. The potential increase in annual net transpiration will most likely change the water balance by negatively affecting soil moisture and hydrological flows from these broadleaved TMFs in summers, and to a lesser degree in winters.

The impacts on carbon assimilation rates of projected increases in atmospheric CO_2_ and associated fertilization effects together with increases in temperature and changes in precipitation will vary between species groups. The pioneer species are likely to maintain high photosynthesis rates by optimizing leaf-specific conductivity, which is the relationship between the conducting area and the total leaf area (Sobrado, 2003). Interestingly, Eckert *et al.* (2021) have shown that late-successional species have the highest rates of refixation of respiratory CO_2_ owing to relatively higher mesophyll resistance and thicker cell walls, which allows them to fix more CO_2_ with fewer open stomata and losing less water. Irrespective of the species groups, the CO_2_ fertilization effect could induce lower moisture uptake as the same amount of carbon can be fixed with fewer stomata open or for less time (Mengis *et al.*, 2015; Yang *et al.*, 2016; Hashimoto *et al.*, 2019). However, the gain in water-use efficiency with CO_2_ fertilization could be offset by higher nocturnal transpiration in secondary forests (Marks and Lechowicz, 2007). Further, the CO_2_ fertilization effect could be limited by the availability of soil nutrients, especially nitrogen and phosphorus, in poorly developed and highly leached mountain soils, such as at our study site (dos Santos *et al.*, 2019; Terrer *et al.*, 2019). Taken together, the postulated increase in productivity with increases in temperature and precipitation could lead to the greening of the TMFs in Sikkim Himalaya, at least in the short term (Sebastian *et al.*, 2019). Similar evidence has already been reported from the wetter parts of eastern Nepal Himalaya, where climate warming and increased summer precipitation are likely to remove moisture constraints on photosynthesis in treeline conifer and broadleaved species (Pandey *et al.*, 2020). Over longer time scales, the increase in productivity might not necessarily lead to higher carbon sequestration and net carbon gain. In a growth simulation study of 141 temperate tree species, Bugmann and Bigler (2011) showed that the higher productivity due to the CO_2_ fertilization effect is associated with proportionately less biomass gain as well as reduced longevity, leading to net zero carbon gains.

### Conclusions

It is still challenging to predict plant water relations in diverse secondary TMFs because of the variability among different functional groups and the diversity in the traits that regulate the relations (Huc *et al.*, 1994; Nogueira *et al.*, 2004; Küppers *et al.*, 2008). In this study, using limited resources, the plant water relations in an East Himalayan wet tropical montane broadleaved forest have been estimated for the first time using methods covering the maximum variability of sap flow. Sap flow was found to be 3–9 times higher than in conspecific species in relatively drier regions of Central Himalaya and East Asia, highlighting the interactive role of precipitation and elevation in modulating the available energy and moisture, and hence plant productivity (Bruijnzeel *et al.*, 2011). Our use of radial and azimuthal probes has opened a whole new dimension of understanding of plant water use for Himalayan species, and their use is highly recommended to minimize the errors in estimating whole-tree sap flow (Shinohara *et al.*, 2013; Komatsu *et al.*, 2016). Our finding of significant nocturnal sap flow, dominated by the pre-dawn period, is the first report from the Himalaya and we postulate two potential roles that might have led to adaptation of pre-dawn sap flux in such wet, energy-limited montane systems: first, to facilitate carbon fixation during the cloud free, early morning hours by ensuring adequate leaf water balance and keeping stomata open, and second to ensure nutrient and O_2_ transport for early-morning developmental activity. Our observations of nocturnal sap flow hold significance for future SPAC work in terms of shifting the timing of pre-dawn leaf water potential measurements (Kavanagh *et al.*, 2007), estimating water use efficiency across species (Chaves *et al.*, 2016), quantifying hydrological services (Siddiq and Cao, 2018; Kangur *et al.*, 2021), and improving regional climate change modelling exercises (De Dios *et al.*, 2015). Other adaptations such as midday depression in shallow-rooted pioneer species highlight their sensitivity to environmental extremes (dos Santos *et al.*, 2019).

Studies on climate change in the Eastern Himalaya have predicted an increase in summer rainfall, declining winter rains, and increasing summer and winter temperatures (Kulkarni *et al.*, 2013; Krishnan *et al.*, 2019). Drier and warmer winters are likely to affect the phenology and negatively affect the productivity of shallow-rooted pioneers such as *S. racemosa* and *E. acuminata*. In contrast, deep-rooted species such as *C. hystrix* and Himalayan oaks might remain unaffected by seasonal droughts (Kumar *et al.*, 2022, Preprint). Thus, secondary broadleaved forests, with a dominance of shallow-rooted pioneers, are more prone to the negative impacts of drier and warmer winters than primary forests, which are dominated by deep-rooted species. In the summer, the onset of rain currently provides enough moisture to ensure peak vegetation productivity in April; however, increased summer precipitation in the future could result in higher cloud cover, negatively affecting both vegetation productivity and transpiration (Donohue *et al.*, 2017), but it might also allow the opportunistic fast-growing pioneers to grow stronger alongside the late-successional species in the short term (Lyu *et al.*, 2020). The CO_2_ fertilization effect could potentially benefit both these species groups, but this could be limited by the availability of soil nutrients such as nitrogen and phosphorus, and any gains are likely to be offset by lower biomass gains and reduced longevity. The net effect of the postulated increase in productivity due to warming, CO_2_ fertilization, and increased precipitation could lead to the greening of the TMFs in Sikkim Himalaya, at least in the short term (Krishnaswamy *et al.*, 2014; Sebastian *et al.*, 2019).

The overall effects of the changes in temperature and precipitation on the biodiversity of the region will be complex and their prediction will require ecohydrological models specific to the East Himalayan TMFs. More regional studies, including diel and seasonal variability in transpiration, will be critical for improving the accuracy of land-surface interaction models and predicting the impact of climate change on Himalayan ecohydrology (Wang and Dickinson, 2012; Miller *et al.*, 2018). Our study provides the first empirical understanding of climatic controls on vegetation water use from a wet, high-elevation tropical broadleaved evergreen montane forest in Eastern Himalaya, one of the 30 global biodiversity hotspots and 200 ecoregions considered to be of importance (Chettri *et al*., 2008). Our GLS model highlighted the interacting roles of *R*_sol_ and VPD in modulating sap flow, and our study contributes to the currently scant literature on plant water relations from secondary TMFs, which have distinct water use behaviour in contrast to primary forests. Our study also highlights the pitfalls of generalizing water use across functional groups, forest types, and climatic conditions (Berdanier *et al.*, 2016), whilst comparing plant water use strategies between co-occurring pioneer and late-successional species with a view to assessing the impacts of future changes in energy and soil moisture conditions (Bretfeld *et al.*, 2018; Moore *et al.*, 2018). Based on our findings, we recommend prioritizing the conservation and management of these secondary forests in the Eastern Himalaya for sustained ecosystem services.

## Supplementary data

The following supplementary data are available at *JXB* online.

Fig. S1. Tree densities of top seven species per hectare at the study site and their basal areas per hectare.

Fig. S2. Time-series plots of the raw daily data for the environmental variables and calculated sap flows of the three species during the study period.

Fig. S3. Variability in wood moisture and density with depth from the cambium in the three study species.

Fig. S4. Scatterplots of sap-flux density versus wood moisture and wood density for sample taken from the three species in December.

Fig. S5. Diel variability in sap flow across the three species as indicated by the correction factor *C*_h_, the sap-flow ratio *R*_NS_.

Fig. S6. Scatterplots of sap flow versus Incoming short-wave radiation and vapour pressure deficit for the three species.

Fig. S7. Scatterplots of nocturnal sap flow versus vapour pressure deficit, soil moisture, and wind velocity for the three species.

Fig. S8. Seasonal variations in daily maximum sap-flux density with depth of the xylem for the three species.

Fig. S9. Boxplots of the seasonal variation in percentage contribution of sap flow at different sapwood depths to daily whole-tree sap flow for the three species.

Fig. S10. Boxplots showing monthly variability in *R*_NS_ as a parameter of azimuthal variability in sap flux density.

Fig. S11. Monthly variability in the contribution of nocturnal sap flow to daily sap flow.

Fig. S12. Comparison of diel patterns between observed whole-tree sap flow and predictions for the three species based on GLS linear regression models.

Table. S1. Slopes and *r*^2^ values of the linear regression models without intercept developed for estimating sap flow in replicate trees using Equation 3.

Table. S2. Results from multiple linear regression models for the day and night periods to assess the drivers of radial variability in sap flow across the three species.

Table. S3. Results from multiple linear regression models for the day and night periods to assess the drivers of azimuthal variability across the three species.

Table. S4. Results from multiple linear regression models to assess the drivers of nocturnal sap flow across the three species.

erad207_suppl_supplementary_figures_S1-S12_tables_S1-S5

## Data Availability

The data supporting the findings of this study are available from the corresponding author, Manish Kumar, on request. A sample subset of the data supporting the findings of this study is also available in the Dryad Digital Repository at https://doi.org/10.5061/dryad.47d7wm3cg; Kumar *et al.* (2023).

## Abbreviations

ACF: autocorrelation coefficient
corARMA: second-order autoregressive moving average
CV: coefficient of variability
GLS: generalized least squares
KGE: Kling–Gupta efficiency
MAP: mean annual precipitation
masl: meters above sea level
MLRM: multiple linear regression models
SPAC: soil–plant–atmosphere continuum
TDP: thermal dissipation probes
TMF: tropical montane forests
VPD: vapour pressure deficit
