# Supplementary material for: Contrasting sap flow characteristics between pioneer and late-successional tree species in secondary tropical montane forests of Eastern Himalaya, India
Source: J Exp Bot. 2023 Jun 8;74(17):5273–93. doi: 10.1093/jxb/erad207 (PMC10498023; doi:10.1093/jxb/erad207)
Supplement: erad207_suppl_supplementary_figures_S1-S12_tables_S1-S5 [file erad207_suppl_supplementary_figures_s1-s12_tables_s1-s5.pdf]

Supplementary data

Figures

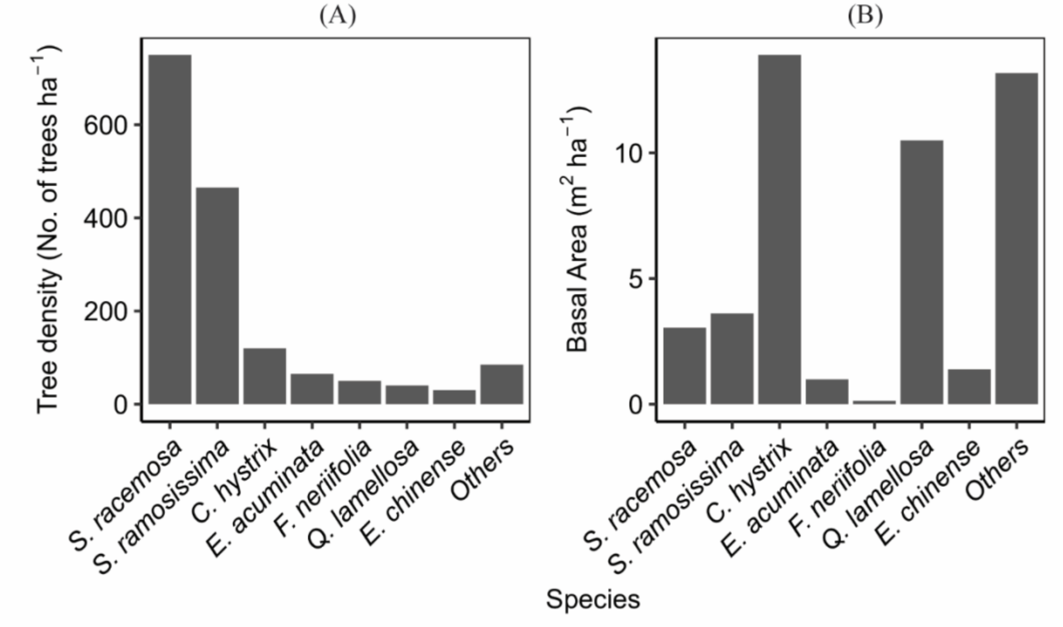

Supplemental Figure S1. Barplots of vegetation distribution in FWS showing (A) tree density of the top seven species per hectare and (B) basal area (m^2 ha^{-1}) of the same species. The selected species, *S. racemosa* and *E. acuminata* represent the fast-growing pioneer community, whereas *C. hystrix* represents the large-sized emergent individuals of the Fagaceae family in the secondary forest stand.

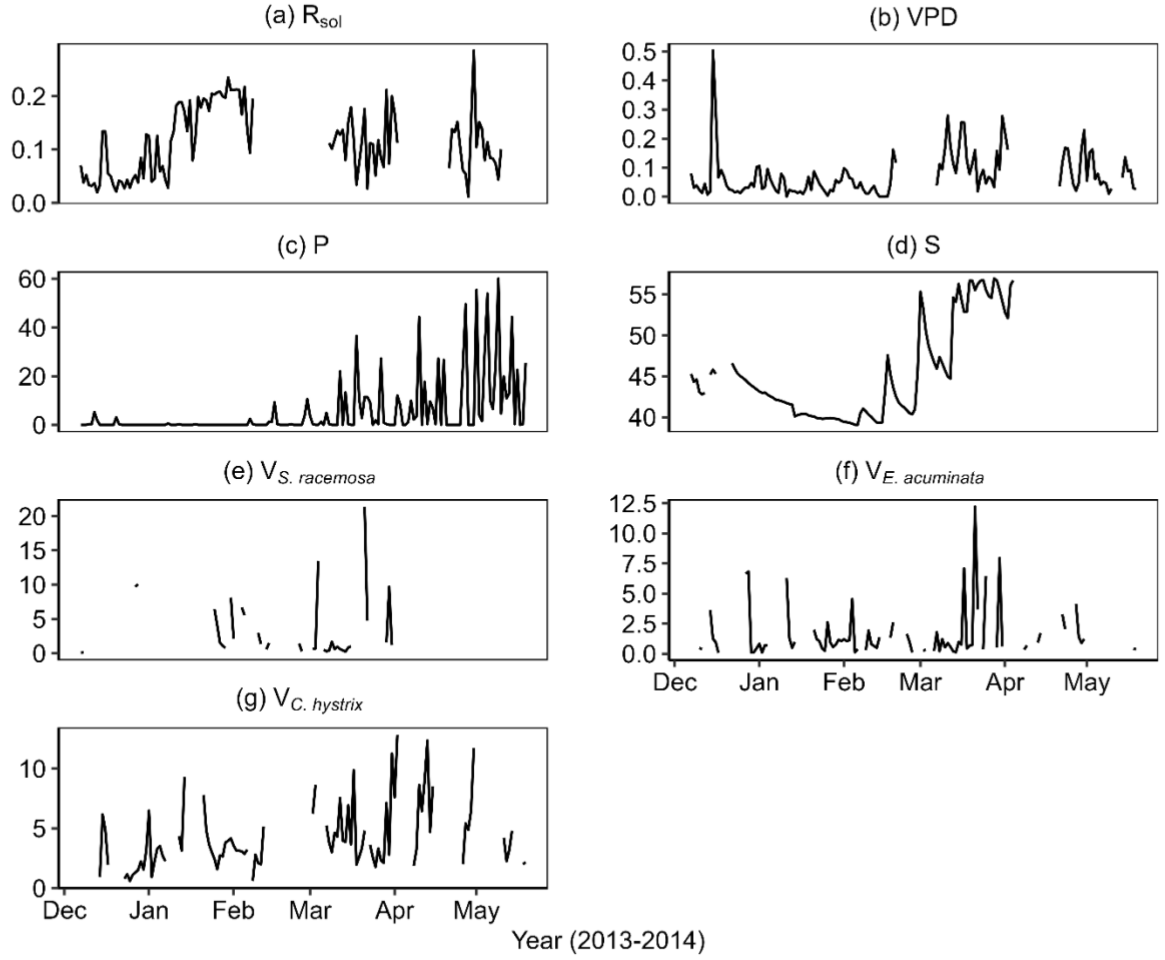

Supplemental Figure S2. Time-series plots of the raw daily data of the SPAC variables from winters (December) to summers (May): (A) Incoming short-wave radiation (R_{sol}, kW m^{-2}), (B) Vapour Pressure Deficit (VPD, kPa), (C) Precipitation (P, mm d^{-1}), (D) Total Soil moisture (S, mm), (E) $V_{S.\ racemosa}$, (F) $V_{E.\ acuminata}$, and (G) $V_{C.\ hystrix}$ are whole-tree sap flow rate (V, kg h^{-1}) of *S. racemosa*, *E. acuminata* and *C. hystrix*, respectively. Maintaining the field instrumentation for a long duration was challenging due to very wet conditions and power failure, and data gaps were observed.

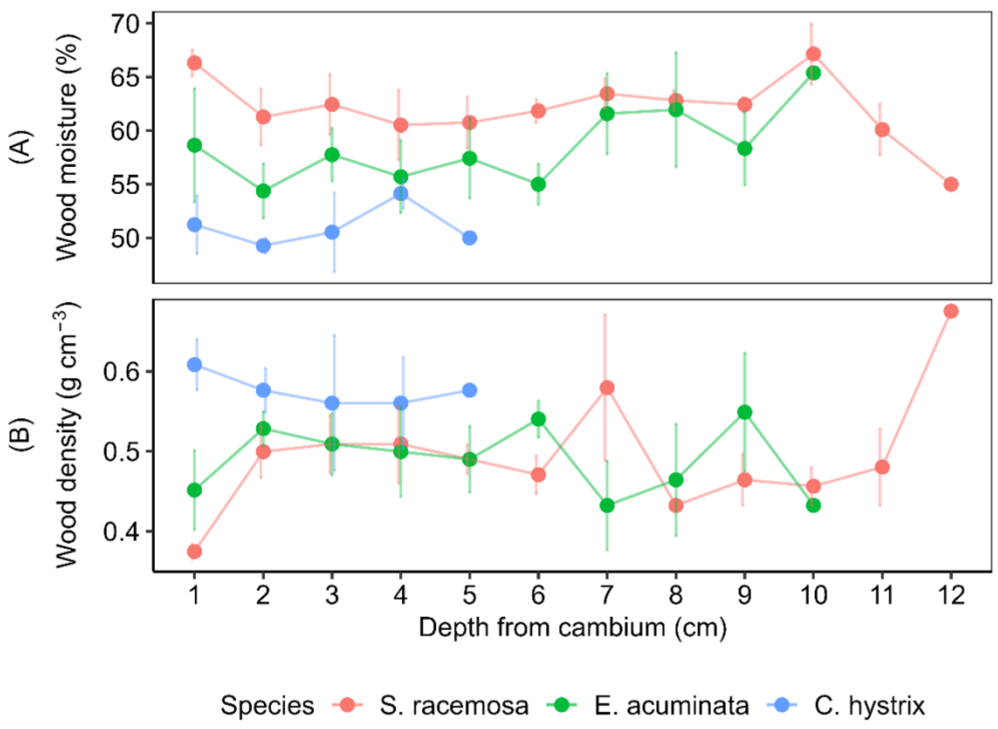

Supplemental Figure S3. Variability in sapwood properties along the depth of xylem in terms of (A) Wood moisture (in %) and (B) Wood density (g cm^{-3}) for the three species. Both, *S. racemosa* and *E. acuminata* show a decline in wood moisture and an increase in wood density from outer to inner xylems, except *C. hystrix*, which shows the highest moisture and lowest wood density in the inner xylem at the 4th cm depth of sapwood.

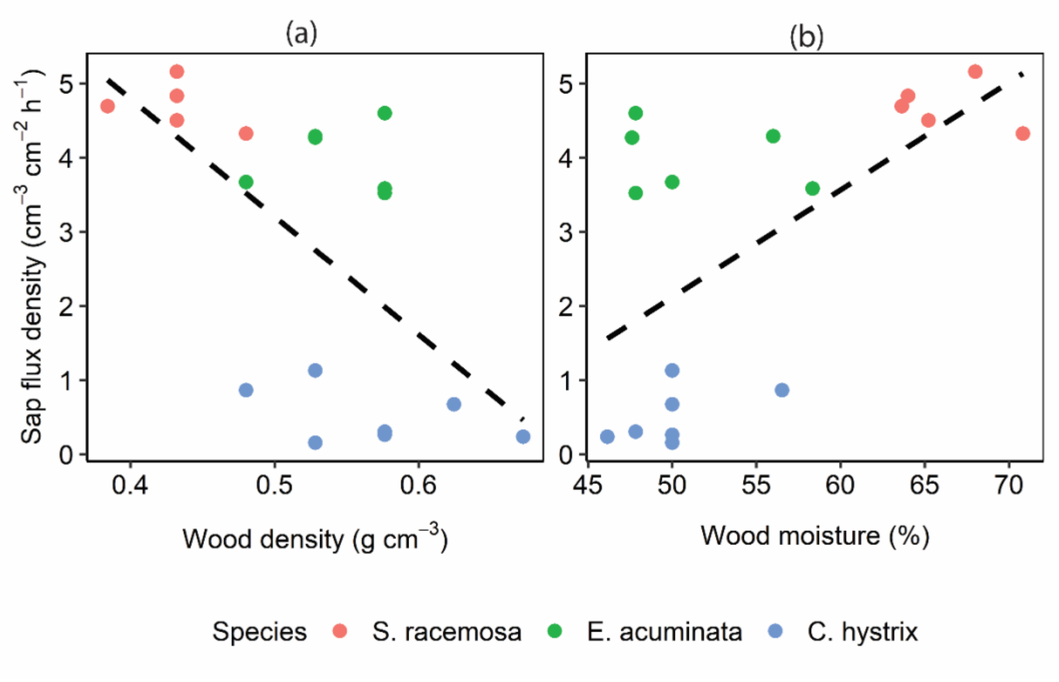

Supplemental Figure S4. Scatterplots showing the relationship between mean hourly sap flux density for 14 December 2013 (when the wood cores were extracted) and (A) Wood moisture (in %) and (B) Wood density (g cm^{-3}) for each sapwood annulus ring in the three species. The dashed line represents the slope of the fitted linear regression model. Both *S. racemosa* and *C. hystrix* showed weakly positive and negative relationships between sap flux density and wood moisture with wood density for a particular sapwood annulus ring.

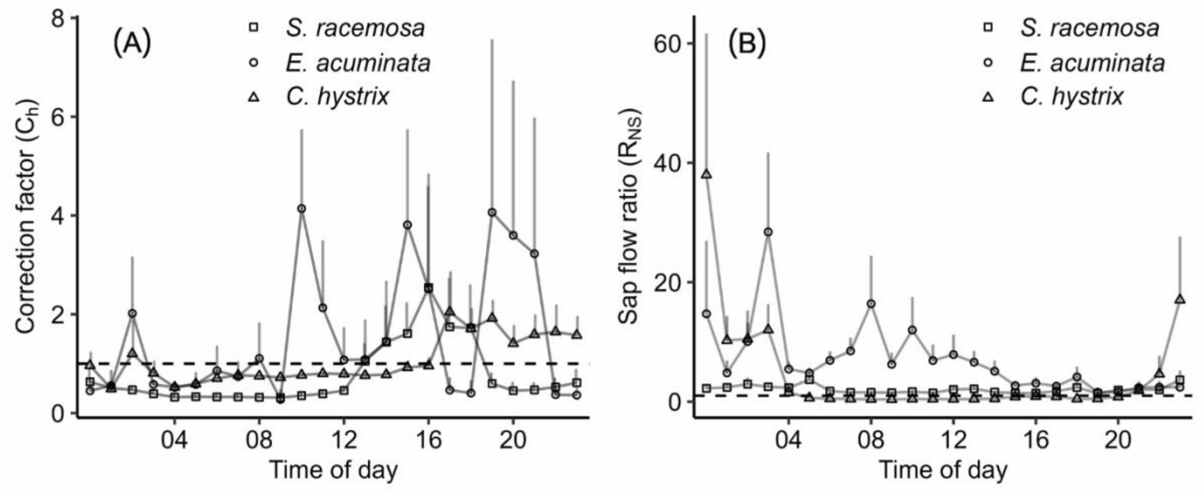

Supplemental Figure S5. Diel variability in sap flow across the three species in (A) diurnal patterns in correction factor (C_h, unitless) used as a proxy of radial variability, (B) diurnal patterns in sap flow ratios (R_{NS}, unitless) used as a proxy of azimuthal variability. The horizontal dotted lines mark the ideal value (1), and error bars represent standard errors.

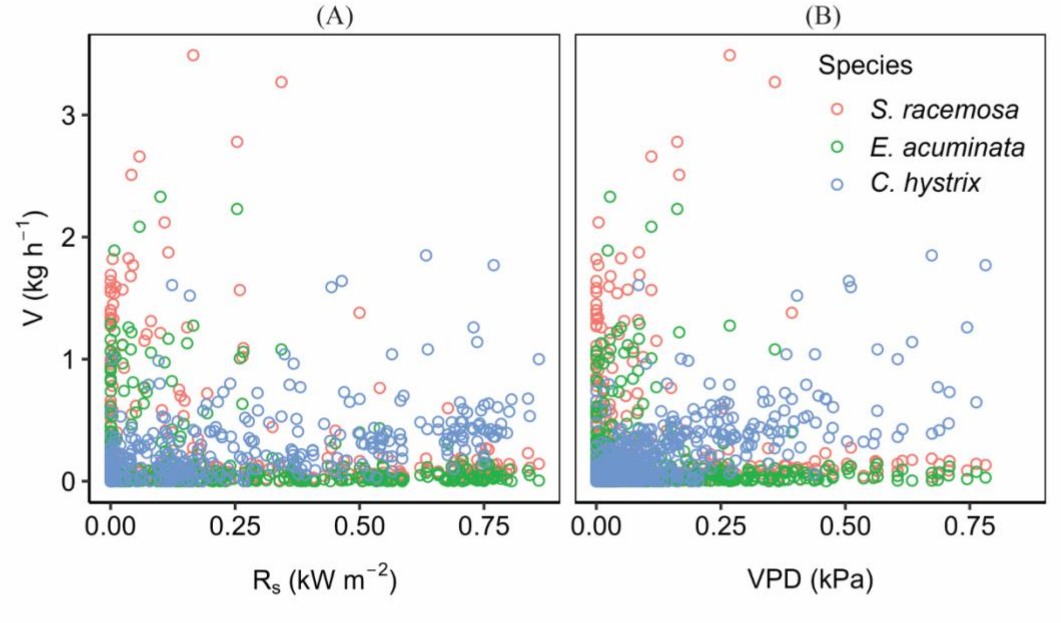

Supplemental Figure S6. Scatterplots between sap flow (V, kg hr^{-1}) and (A) Incoming short-wave radiation (R_s, kW m^{-2}) and (B) Vapour pressure deficit (VPD, kPa) for the three species. Peak sap flow values in *S. racemosa* and *E. acuminata* were concentrated at low R_s and VPD, whereas *C. hystrix* was unaffected by environmental extremes.

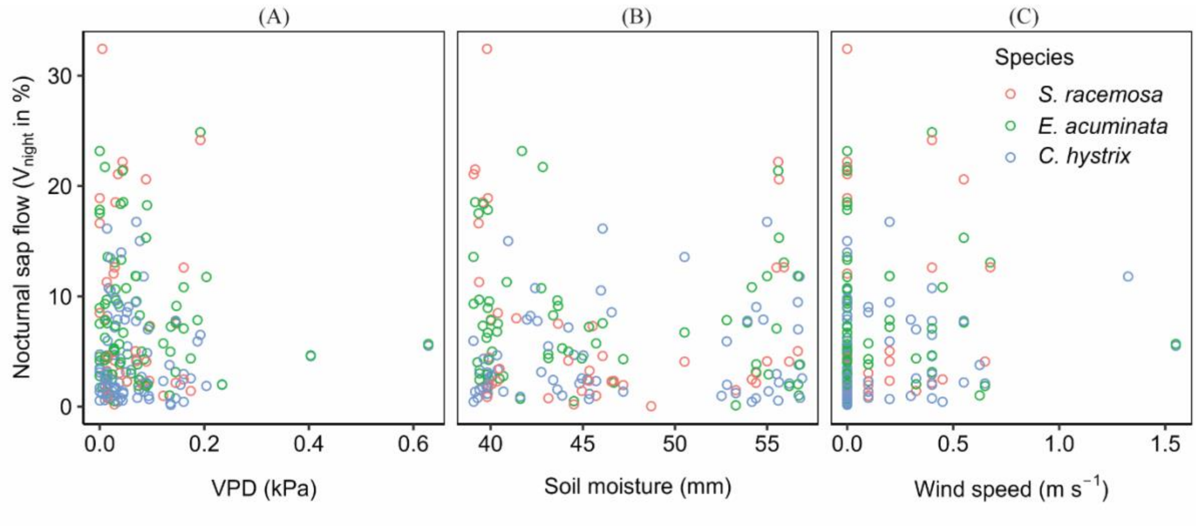

Supplemental Figure S7. Scatterplots between nocturnal sap flow (V_{night} in % of daily V) and (A) Vapour pressure deficit (VPD), (B) Soil moisture (S) and (C) Wind velocity (u) across the three species. Nocturnal sap flow was associated with low VPD (< 0.1 kPa), low wind velocity (<0.5 m s^{-1}) and moderately saturated soil moisture values.

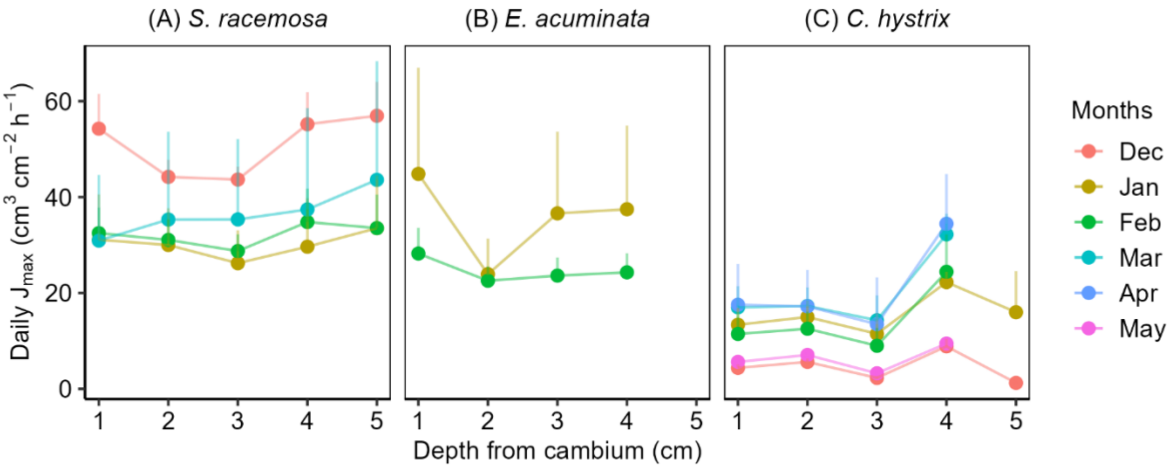

Supplemental Figure S8. Radial variability in sap flux density across different seasons is shown by plotting the average daily maximum sap flux density (Daily J_{max}, cm^3 cm^{-2} h^{-1}) along the depth of the xylem (the error bars represent standard errors).

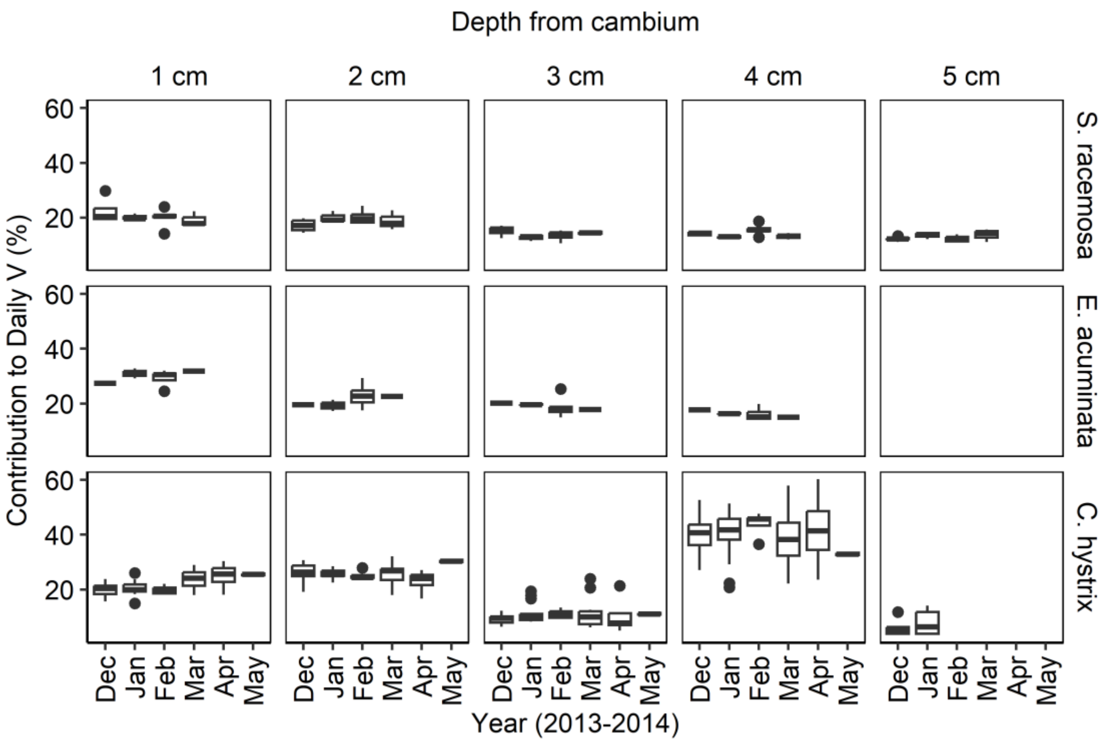

Supplemental Figure S9. Boxplots of the percentage contribution of sap flow at different sapwood depths to daily whole-tree sap flow (V, kg h^{-1}) plotted against different months for the three species.

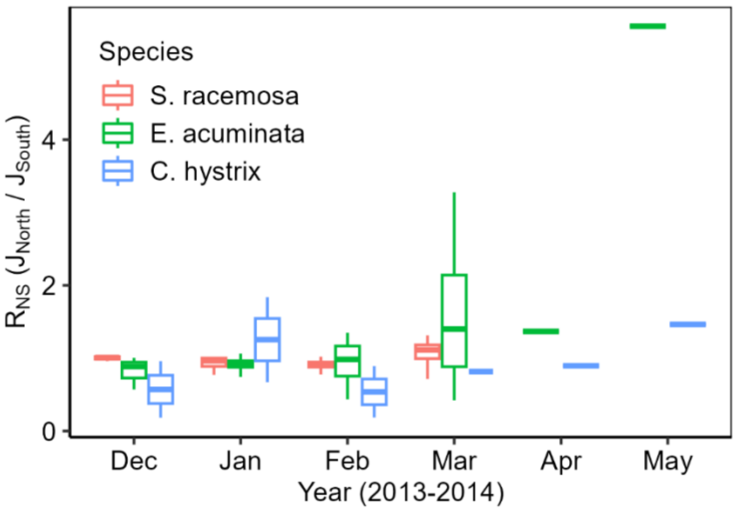

Supplemental Figure S10. Boxplots showing variability in R_{NS} as a parameter of azimuthal variability in sap flux density across the different months for the three species.

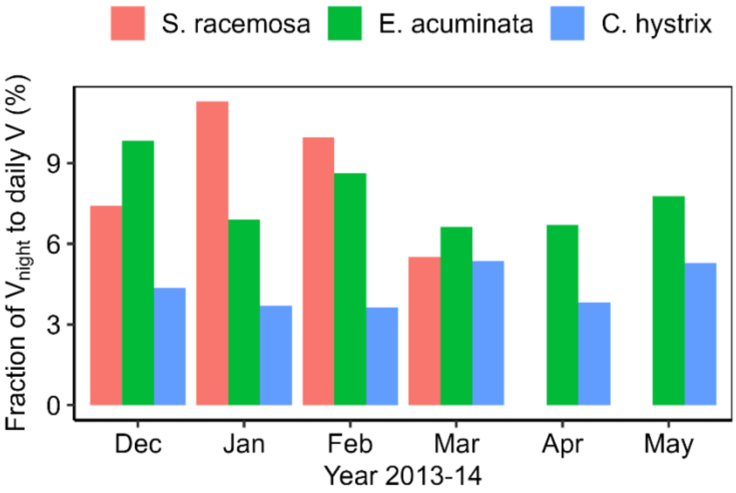

Supplemental Figure S11. Barplot showing variability in the contribution of nocturnal sap flow to daily sap flow across the different months for the three species.

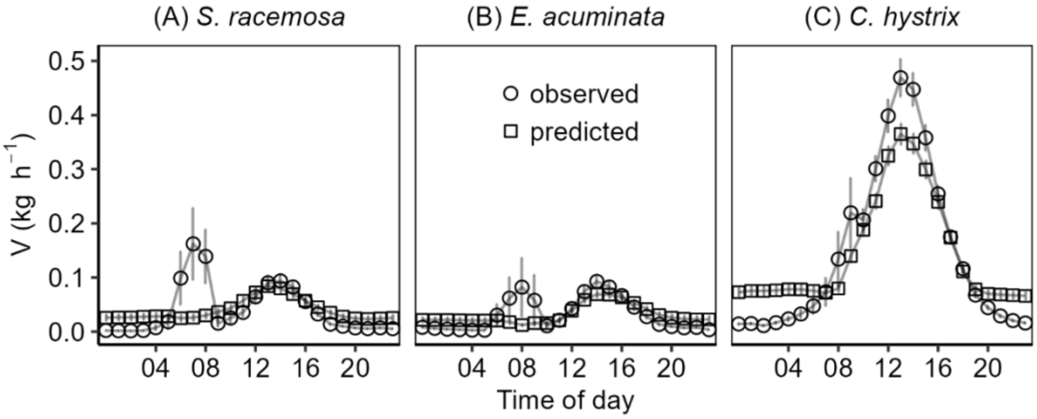

Supplemental Figure S12. Comparison of diurnal patterns in observed and predicted whole-tree sap flow (V, kg h^{-1}) of (A) *S. racemosa*, (B) *E. acuminata* and (C) *C. hystrix* derived from the Generalized least squares (GLS) linear regression models for the three species (Error bars denote standard errors).

Tables

Table S1A. Number of sample days for each tree per species (*n*) used in Fig. 2 after quality checks used for final analysis.

**Tree number**	***S. racemosa***	***E. acuminata***	***C. hystrix***
Tree 1	10	5	28
Tree 2	20	22	19
Tree 3	27	19	18
Tree 4	20	19	NA
Tree 5	23	15	NA

Table S1B. Number of sample days for each tree per species (*n*) used in Fig. 3 after quality checks used for final analysis.

**Tree number**	***S. racemosa***	***E. acuminata***	***C. hystrix***
Tree 1	16	11	99
Tree 2	35	35	60
Tree 3	42	30	47
Tree 4	33	66	NA
Tree 5	38	22	NA

Table S2. Slopes (m) and r^2 (in bracket) of the linear regression models without intercept (LRMs) developed for each hour of the day for the three species *S. racemosa*, *E. acuminata*, and *C. hystrix* used for estimating sap flow in replicate trees using equation 3.

Hours	*S. racemosa* (m (r^2))	*E. acuminata* (m (r^2))	*C. hystrix* (m (r^2))
0	0.59 (0.47)	1.13 (0.92)	0.96 (0.81)
1	0.53 (0.67)	0.96 (0.98)	0.86 (0.67)
2	0.55 (0.85)	0.98 (0.99)	0.6 (0.62)
3	0.66 (0.97)	1.01 (0.99)	0.7 (0.96)
4	0.74 (0.99)	0.93 (0.99)	0.78 (0.99)
5	0.79 (0.99)	0.94 (0.99)	0.82 (0.99)
6	0.87 (0.99)	0.99 (0.98)	0.83 (0.99)
7	0.9 (1)	1.08 (0.97)	0.84 (0.99)
8	0.91 (0.99)	1.16 (0.98)	0.86 (0.99)
9	0.86 (0.99)	1.05 (1)	0.86 (0.99)
10	0.76 (0.97)	0.7 (0.9)	0.78 (0.98)
11	0.77 (0.95)	0.69 (0.89)	0.83 (0.95)
12	0.87 (0.98)	0.78 (0.86)	0.92 (0.96)
13	0.96 (0.98)	0.95 (0.81)	0.96 (0.96)
14	1.07 (0.98)	1.01 (0.97)	0.95 (0.97)
15	1.09 (0.98)	1 (0.99)	0.92 (0.98)
16	1 (0.98)	1.01 (0.98)	0.96 (0.97)
17	0.89 (0.95)	0.88 (0.98)	0.96 (0.97)
18	0.61 (0.88)	0.95 (0.72)	0.96 (0.97)
19	0.63 (0.83)	0.77 (0.59)	0.92 (0.94)
20	0.61 (0.62)	0.69 (0.53)	0.86 (0.91)
21	0.66 (0.51)	0.72 (0.52)	0.8 (0.85)
22	1.02 (0.87)	0.69 (0.5)	0.76 (0.81)
23	1 (0.97)	0.72 (0.46)	0.74 (0.75)

Table S3. Results from multiple linear regression models (MLRMs) for the day (06.00-18.00 h) and the night (18.00-06.00 h) periods to assess the drivers of radial variability in sap flow across the three species using correction factors (C_h) as the response variable and Vapour pressure deficit (VPD, kPa), Incoming short-wave radiation (R_s, kW m^{-2}) and Soil moisture (S, mm) as predictors (**P*<0.05, ** *P*<0.01, *** *P*<0.001). R_s and S were significant predictors of daytime radial variability (C_h, unitless) in *S. racemosa,* whereas none of the predictors was significant for *E. acuminata* and *C. hystrix*. Conversely, VPD was a strong negative predictor of night C_h in *E. acuminata* and a weak negative predictor in *S. racemosa* and *C. hystrix*.

**Species**	**Model**	**Predictors**	**Estimate**	**Std. Error**	**t value**	**p-value (*P*)**	**r^2**
S. racemosa	Day	VPD	0.083	0.268	0.309	0.759	0.43
		**R_s**	**-0.452**	**0.115**	**-3.923**	**<0.001*****	
		**S**	**1.446**	**0.210**	**6.879**	**<0.001*****	
	Night	VPD	-6.766	5.140	-1.316	0.192	0.02
		S	-0.024	1.213	-0.020	0.984	
E. acuminata	Day	VPD	-2.580	6.269	-0.412	0.683	0.007
		R_s	0.693	1.846	0.376	0.710	
		S	0.370	5.471	0.068	0.947	
	Night	**VPD**	**-61.94**	**13.280**	**-4.664**	**<0.001*****	0.40
		S	2.683	7.110	0.377	0.708	
C. hystrix	Day	VPD	1.186	1.102	1.076	0.283	0.01
		R_s	-0.634	0.437	-1.452	0.148	
		S	0.108	0.824	0.131	0.896	
	Night	VPD	-1.429	3.359	-0.425	0.671	0.001
		S	-0.081	0.688	-0.117	0.907	

Table S4. Results from multiple linear regression models (MLRMs) for the day (06.00-18.00 h) and the night (18.00-06.00 h) periods to assess the drivers of azimuthal variability across the three species using sap flow ratio (R_{NS}, unitless) as the response variable and Vapour pressure deficit (VPD, kPa), Incoming short-wave radiation (R_s, kW m^{-2}) and Soil moisture (S, mm) as predictors (*P<0.05, ** P<0.01, *** P<0.001). The MLRMs performed poorly. However, R_s was a significant positive predictor of R_{NS} at average VPD and S across the three species. Night-time R_{NS} was better predicted by VPD and S in *S. racemosa*, and by VPD in *C. hystrix*. S was a significant predictor of night R_{NS} in *E. acuminata*.

**Species**	**Daytime**	**Predictors**	**Estimate**	**Std. Error**	**t value**	**p-value (*P*)**	**r^2**
S. racemosa	Day	VPD	-3.143	4.351	-0.722	0.473	0.05
		**R_s**	**3.299**	**1.872**	**1.762**	**0.083**	
		S	-2.874	3.416	-0.842	0.403	
	Night	VPD	-25.142	13.612	-1.847	0.069	0.09
		**S**	**5.716**	**3.213**	**1.779**	**0.080**	
E. acuminata	Day	VPD	15.209	19.143	0.795	0.433	0.18
		R_s	9.894	5.637	1.755	0.089	
		S	-21.078	16.705	-1.262	0.216	
	Night	VPD	13.409	13.279	1.010	0.320	0.16
		**S**	**15.501**	**7.110**	**2.180**	**0.037***	
C. hystrix	Day	VPD	23.524	41.535	0.566	0.572	0.03
		**R_s**	**-34.519**	**16.551**	**-2.086**	**0.038***	
		S	33.430	31.089	1.075	0.284	
	Night	**VPD**	**4.454**	**1.517**	**2.937**	**0.004****	0.05
		S	0.036	0.311	0.117	0.907	

Table S5. Results from multiple linear regression models (MLRMs) to assess the drivers of nocturnal sap flow (V_{night}) across the three species measured as a percentage fraction of daily sap flow as the response variable and Vapour pressure deficit (VPD, kPa), Wind speed (U, m s^{-1}) and Soil moisture (S, mm) as predictors. S was a significant negative predictor of V_{night} in *E. acuminata*, whereas VPD was a significant negative predictor for *C. hystrix*. None of the predictors was significant for *S. racemosa*.

**Species**	**Predictors**	**Estimate**	**Std. Error**	**t value**	**p-value (*P*)**	**r^2**
	VPD	-0.115	0.345	-0.333	0.742	
S. racemosa	S	-0.002	0.003	-0.647	0.524	0.02
	U	0.059	0.108	0.548	0.589	
	VPD	0.073	0.321	0.228	0.821	
E. acuminata	**S**	**-0.006**	**0.003**	**-2.010**	**0.050**	0.12
	U	0.039	0.098	0.402	0.690	
	**VPD**	**-0.382**	**0.141**	**-2.715**	**0.009**	
C. hystrix	S	0.001	0.001	0.588	0.559	0.13
	U	0.015	0.030	0.499	0.620	
